# Asymptotic behavior of an intrinsic rank-based estimator of the Pickands dependence function constructed from B-splines

**DOI:** 10.1007/s10687-022-00451-9

**Published:** 2022-11-09

**Authors:** Axel Bücher, Christian Genest, Richard A. Lockhart, Johanna G. Nešlehová

**Affiliations:** 1 Heinrich-Heine-Universität Düsseldorf, Düsseldorf, Germany; 2 McGill University, Montréal, Canada; 3 Simon Fraser University, Burnaby, Canada

**Keywords:** B-spline, Extreme-value copula, Minimum distance estimator, Pickands dependence function, Rank-based inference, Spectral distribution, 60G70, 62G32, 62H10

## Abstract

A bivariate extreme-value copula is characterized by its Pickands dependence function, i.e., a convex function defined on the unit interval satisfying boundary conditions. This paper investigates the large-sample behavior of a nonparametric estimator of this function due to Cormier et al. (Extremes 17:633–659, 2014). These authors showed how to construct this estimator through constrained quadratic median B-spline smoothing of pairs of pseudo-observations derived from a random sample. Their estimator is shown here to exist whatever the order $m \ge 3$ of the B-spline basis, and its consistency is established under minimal conditions. The large-sample distribution of this estimator is also determined under the additional assumption that the underlying Pickands dependence function is a B-spline of given order with a known set of knots.

## Introduction

Let (*X*, *Y*) be a continuous random pair with joint distribution function *H*. Let *F* and *G* denote the margins of *X* and *Y*, respectively. The unique copula *C* characterizing the dependence between *X* and *Y* is then the joint distribution of the pair $(U, V) = (F(X), G(Y))$.

Of frequent interest in applications, e.g., in quantitative risk management, are situations where the underlying copula *C* is unknown but assumed to belong to the class $\mathcal {C}$ of extreme-value copulas. It was shown by Pickands (1981) that $C \in \mathcal {C}$ if and only if, for all $u, v \in (0,1)$,

1 $$\begin{aligned} C(u, v) = \exp \left[ \ln (uv) A \left\{ \frac{\ln (v)}{\ln (uv)}\right\} \right] \end{aligned}$$

for a convex map $A: [0, 1] \rightarrow [1/2,1]$ such that, for all $t \in [0,1]$, $\max (t, 1-t ) \le A(t) \le 1$. The map *A* is called a Pickands dependence function and the class of such functions is hereafter denoted $\mathcal {A}$.

Given a random sample from *H*, one can estimate *C* readily by plugging into (1) a nonparametric estimator $A_n$ of *A*. This leads to a bona-fide extreme-value copula so long as $A_n$ is intrinsic, i.e., $A_n \in \mathcal {A}$. Estimation of *A* has already been considered, both when the margins *F* and *G* are known, and in the more realistic case where they are not. See, e.g., Gudendorf and Segers (2010) or Genest and Nešlehová (2012) for surveys of the early literature.

Major contributions to this problem in recent years include Berghaus et al. (2013), Peng et al. (2013), Cormier et al. (2014), Ferreira (2017), Marcon et al. (2017), and Escobar-Bach et al. (2018). A vast simulation-based comparison of various estimators was performed by Vettori et al. (2018).

The purpose of this paper is to study, for the first time, the large-sample behavior of a rank-based, intrinsic estimator of *A* based on constrained B-spline smoothing (Cormier et al. 2014). From the work of these authors and Vettori et al. (2018), this estimator is known to perform well in finite-sample settings when compared, e.g., to the madogram-based procedure of Naveau et al. (2009) and to the more traditional Pickands, CFG, and Hall–Tajvidi estimators made intrinsic via the technique of Fils-Villetard et al. (2008).

An extended version of the estimator proposed by Cormier et al. (2014) is introduced in Sect. 2. Its existence is established for B-splines of any order $m \ge 3$ in Sect. 3, and conditions under which it is consistent are detailed in Sect. 4. The proof of this result, Theorem 1, is then given in Sect. 5.

It seems difficult to determine the asymptotic distribution of the estimator of Cormier et al. (2014) in full generality. As described in Sect. 6, however, it is possible to achieve this goal in the much more restrictive—but nonetheless instructive—case where the unknown underlying Pickands dependence function *A* is a B-spline of given order *m* with *k* known internal knots, i.e., *A* can be expressed as a linear combination

2 $$\begin{aligned} A = \beta _1 \phi _{1,m} + \cdots + \beta _{m+k} \phi _{m+k,m}, \end{aligned}$$

of $m + k$ B-spline basis elements that are piece-wise polynomials of degree $m-1$ on a partition of (0, 1) induced by *k* fixed points called internal knots.

The large-sample distribution of the estimator ${\hat{\beta }}_n$ of $\beta$ is given in Sect. 6 for B-splines of degree $m \in \{ 3, 4 \}$. The proof of this convergence result, Theorem 2, is detailed in Sect. 7. The large-sample distribution of ${\hat{\beta }}_n$ is then used in Sect. 8 to establish the asymptotic behavior of ${\hat{A}}_n = {\hat{\beta }}_n ^\top \mathit{\Phi}$ and of its first and second derivatives. As explained there, the latter lead to consistent and asymptotically unbiased estimators of the spectral distribution and density.

The assumption that a Pickands dependence function is of the form (2) is fairly mild, given that any convex function can be approximated with any desired accuracy by increasing the number of internal knots (de Boor 1978). What is restrictive is the assumption that the knots can be identified in advance. While weakening this assumption is beyond the scope of the present paper, Theorem 1 suggests that this should be possible and Theorem 2 provides useful information on the properties and shape that the limit will take.

## Construction of an intrinsic B-spline estimator of *A*

Let $(X_1, Y_1), \ldots , (X_n,Y_n)$ be a random sample from the unknown joint continuous distribution *H* with underlying copula *C* and, for every real $t \in \mathbb {R}$, set

$$F_n (t) = \frac{1}{n} \sum _{i=1}^n \mathbf {1}(X_i \le t), \quad G_n (t) = \frac{1}{n} \sum _{i=1}^n \mathbf {1}(Y_i \le t),$$

where $\mathbf {1}(E)$ denotes the indicator function of the set *E*. For each integer $i \in \{ 1, \ldots , n \}$, write

$$\hat{U}_i = F_n (X_i), \quad \hat{V}_i = G_n(Y_i ),$$

and let ${\hat{C}}_n$ be the empirical copula defined, for all real $u, v \in [0,1]$, by

3 $$\begin{aligned} {\hat{C}}_n (u, v) = \frac{1}{n} \sum _{i=1}^n \mathbf {1} ({\hat{U}}_i \le u, {\hat{V}}_i \le v). \end{aligned}$$

Suppose that *C* is of the form (1) for some unknown Pickands dependence function *A*. To construct an intrinsic nonparametric estimator of *A* using *B*-splines, first observe as in Cormier et al. (2014) that, for any real $u, v \in (0,1)$,

$$t = \frac{\ln (v)}{\ln (uv)} \quad \Rightarrow \quad A (t) = \frac{\ln \{ C (u, v)\}}{\ln (uv)} \, .$$

Thus if one sets, for each integer $i \in \{1, \ldots , n \}$,

4 $$\begin{aligned} {\hat{T}}_i = \frac{\ln (\hat{V}_i)}{\ln (\hat{U}_i\hat{V}_i)} \, , \quad {\hat{Z}}_i = \frac{\ln \{ {\hat{C}}_n (\hat{U}_i,\hat{V}_i)\}}{\ln (\hat{U}_i \hat{V}_i)} \, , \end{aligned}$$

the pairs $({\hat{T}}_1, {\hat{Z}}_1), \ldots , ({\hat{T}}_n, {\hat{Z}}_n)$ should lie on, or near, the curve $t \mapsto A (t)$, given that ${\hat{C}}_n$ is a uniformly strongly consistent estimator of *C* (Gänßler and Stute 1987; Genest et al. 2017). A graph of these pairs is termed an *A*-plot in Cormier et al. (2014).

To estimate *A*, Cormier et al. (2014) propose to fit through the pairs $({\hat{T}}_1, {\hat{Z}}_1), \ldots , ({\hat{T}}_n, {\hat{Z}}_n)$ a linear combination ${\hat{A}}_n$ of B-splines of order 3 under shape constraints which ensure that ${\hat{A}}_n \in \mathcal {A}$. Their approach, which extends readily to B-splines of arbitrary order $m \ge 3$, is described below.

### Definition of B-splines

For any given integer $m \ge 2$, consider a partition on the interval (0, 1) induced by *k* points, viz.

5 $$\begin{aligned} 0< \tau _{m+1}< \cdots< \tau _{m+k} < 1 \end{aligned}$$

called internal knots. For notational convenience, further set

$$\tau _1 = \cdots = \tau _m = 0 \quad \text{ and } \quad \tau _{m+k+1} = \cdots = \tau _{2m+k} = 1.$$

The entire sequence $(\tau _1, \ldots , \tau _{2m+k})$ is then denoted $\varvec{\tau }$.

A B-spline of order *m* on the interval [0, 1] with knot sequence $\varvec{\tau }$ is a continuous function which is a polynomial of degree $m-1$ when restricted to any interval of the form $(\tau _{m+j}, \tau _{m+j+1})$ with $j \in \{ 0, \ldots , k \}$. Any B-spline can be written as a linear combination of $m+k$ orthogonal functions $\phi _{1,m}, \dots , \phi _{m+k,m}$. Each of these so-called B-spline basis functions is a piece-wise polynomial of degree $m-1$ constructed in such a way that its $m - 2$ first derivatives are continuous. Let

6 $$\begin{aligned} \mathit{\Phi} = (\phi _{1,m}, \dots , \phi _{m+k,m})^\top \end{aligned}$$

be the vector of B-spline basis functions corresponding to the set of knots (5). The set of B-splines of order *m* is then given by $\{ \beta ^\top \mathit{\Phi} : \beta \in \mathbb {R}^{m+k} \}$.

### Construction of the B-spline basis

The B-spline basis $\phi _{1,m}, \dots , \phi _{m+k,m}$ of order *m* with *k* distinct internal knots (5) is built iteratively as follows via the Cox–de Boor recursion formula; see, e.g., de Boor (1978). For every integer $j \in \{ 1,\ldots , k+2m-1\}$, let

$$\phi _{j,1} = \mathbf {1} [\tau _j, \tau _{j+1}).$$

Then for each integer $\ell \in \{ 2, \ldots , m \}$, define recursively, for every integer $j \in \{ 1, \ldots , k+2m-\ell \}$ and real $t \in [0, 1]$,

$$\phi _{j,\ell }(t) = \frac{t-\tau _j}{\tau _{j+\ell -1}- \tau _j}\, \phi _{j,\ell -1}(t) + \frac{\tau _{j+\ell }-t}{\tau _{j+\ell }- \tau _{j+1}} \,\phi _{j+1,\ell -1}(t).$$

**Fig. 1:**
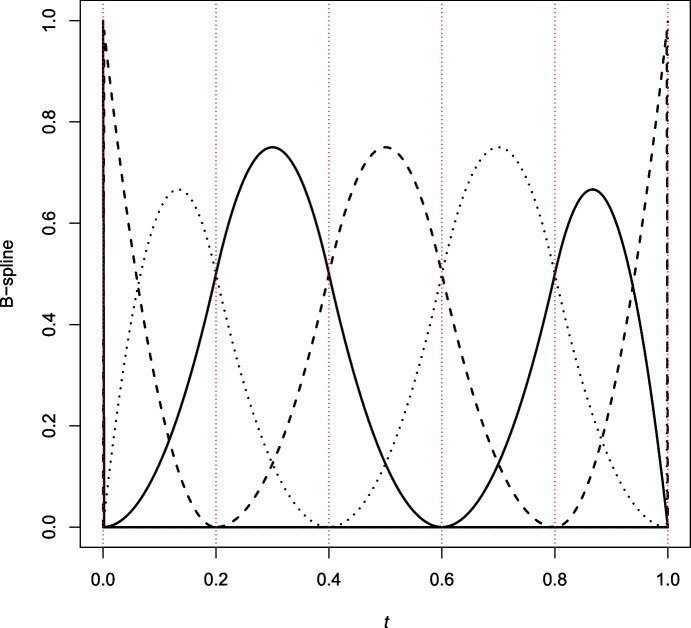
B-spline basis of order $m = 3$ with $k = 4$ equally spaced internal knots consisting of $m+k = 7$ piece-wise polynomials of degree $m-1 = 2$

The result of this construction is illustrated in Fig. 1 in the special case when $m = 3$ and $k = 4$ internal knots. The basis consists of $m + k = 7$ piece-wise polynomials of degree $m - 1 = 2$. The first basis element, $\phi _{1,3}$ (dashed line), is monotone decreasing on (0, 0.2) and zero elsewhere. The second element, $\phi _{2,3}$ (dotted curve), is unimodal on (0, 0.4) and zero elsewhere. The third element, $\phi _{3,3}$ (solid line) is unimodal on (0, 0.6) and zero elsewhere. The 4th (dashed line), 5th (dotted line), and 6th (solid line) basis elements are non-zero on (0.2, 0.8), (0.4, 1), and (0.6, 1), respectively. Finally, $\phi _{7,3}$ (dashed line), vanishes on (0, 0.8) and is monotone increasing on (0.8, 1).

In general, the first basis function $\phi _{1,m}$ takes the value 1 at 0 and decreases monotonically to zero. Analogously, the last basis function $\phi _{m+k, m}$ starts at 0 and rises monotonically to 1, which it reaches at 1. In contrast, each interior basis function $\phi _{2,m}, \ldots , \phi _{m+k-1, m}$ is zero left of a certain internal knot, at which point it rises monotonically to a peak before falling back monotonically to zero, where it remains thereafter. By construction, B-spline basis functions of order *m* are strictly positive over at most *m* adjacent intervals.

### Construction of the B-spline estimator

In the spirit of Cormier et al. (2014), who considered only B-splines of order 3, fix an integer $m \ge 3$ and a set of *k* distinct internal knots (5) with corresponding B-spline basis $\mathit{\Phi}$ of order *m* as defined in (6). Further pick an arbitrary smoothing constant $\lambda _n \in (0, \infty )$, where *n* is the sample size.

A B-spline estimator of unknown underlying Pickands dependence function *A* is then given by

7 $$\begin{aligned} \hat{A}_n = \hat{\beta }_n^\top \mathit{\Phi} , \end{aligned}$$

where ${\hat{\beta }}_n = ({\hat{\beta }}_1, \ldots , {\hat{\beta }}_{m+k})^\top$ is any minimizer of the objective function

8 $$\begin{aligned} L(\beta ) = \Vert {\hat{Z}} - \beta ^\top \mathit{\Phi} ({\hat{T}}) \Vert _1 + \lambda _n \, \Vert \beta ^\top \mathit{\Phi} '' \Vert _{\infty } \end{aligned}$$

based on the vectors ${\hat{T}} = ({\hat{T}}_1, \ldots , {\hat{T}}_n)^\top$ and ${\hat{Z}} = ({\hat{Z}}_1, \ldots , {\hat{Z}}_n)^\top$ of pseudo-observations with components defined in Eq. (4). Here, $\Vert \cdot \Vert _1$ and $\Vert \cdot \Vert _\infty$ refer to the $\ell _1$ (taxicab) and maximum norm, respectively.

Cormier et al. (2014) favored the $\Vert \cdot \Vert _1$ norm over the more standard $\Vert \cdot \Vert _2$ norm for added robustness, in keeping with the median smoothing approach of Koenker et al. (1994) and its implementation in the R package cobs along with the constrained optimization method described by He and Ng (1999).

To ensure shape constraints, the function *L* is minimized over the set

9 $$\begin{aligned} \mathcal {B} = \{ \beta \in \mathbb {R}^{m+k}: \beta ^\top \mathit{\Phi} \in \mathcal {A} \} \end{aligned}$$

of vectors $\beta = (\beta _1, \ldots , \beta _{m+k})^\top$ in $\mathbb {R}^{m+k}$ for which the B-spline

$$\beta ^\top \mathit{\Phi} = \beta _1 \phi _{1,m} + \cdots + \beta _{m+k} \phi _{m+k,m}$$

is a Pickands dependence function. From an operational viewpoint the set $\mathcal {B}$ consists of vectors $\beta \in \mathbb {R}^{m+k}$ that satisfy the following three conditions:

(C1): $$\beta ^\top \mathit{\Phi} (0) = \beta ^\top \mathit{\Phi} (1) = 1$$ or, equivalently, $$\beta _1 = \beta _{m+k} = 1$$.
(C2): $\beta ^\top \mathit{\Phi} ''(t) \ge 0$ for every real $t \in [0, 1]$, with the convention that when the second derivative fails to exist, the inequality holds for either choice of meaningful one-sided derivative.
(C3): $\beta ^\top \mathit{\Phi} ^\prime (0) \ge -1$ and $\beta ^\top \mathit{\Phi} ^\prime (1) \le 1$, where the derivatives are one-sided.

Condition (C1) is the same as in Cormier et al. (2014). When $m \in \{ 3, 4 \}$, Condition (C2) is equivalent to the requirement that

$$\forall _{j \in \{ 0, \ldots , k + 1\}} \quad \beta ^\top \mathit{\Phi} ''(\tau _{m+j}) \ge 0,$$

which ensures that $\beta ^\top \mathit{\Phi}$ is convex and meets the end-point constraints because the second derivative $\beta ^\top \mathit{\Phi} ^{\prime \prime }$ is then linear between the knots, and hence non-negativity at the knots guarantees non-negativity everywhere on [0, 1]. When (C1) and (C2) hold, (C3) guarantees that

$$\forall _{t \in [0,1]} \quad \max (t, 1-t) \le \beta ^\top \mathit{\Phi} (t) \le 1.$$

Accordingly, the estimator is intrinsic, i.e., ${\hat{A}}_n \in \mathcal {A}$.

Note that (C3) is a better and more economical choice than the approximate condition of Cormier et al. (2014), which stated that for some large, unspecified integer *N* and all $j \in \{ 1, \ldots , N-1\}$,

$$\max (j/N, 1-j/N) \le \beta ^\top \mathit{\Phi} (j/N) \le 1.$$

The second summand in Eq. (8) is a penalization term. It plays an important role when the number of knots and their locations are unknown, as is often the case in practice. The minimization procedure is then typically performed over a large number of equally-spaced quantiles of the empirical distribution of the pseudo-sample ${\hat{T}}_1, \ldots , {\hat{T}}_n$. It is well known that this penalization can be expressed equivalently as a set of constraints on the coefficients, viz.

$$|\beta ^\top \mathit{\Phi} ^{\prime \prime }(\tau _{j+1}) - \beta ^\top \mathit{\Phi} ^{\prime \prime }(\tau _{j})|\le \lambda ^*,$$

which must hold for some $\lambda ^*$ and every integer $j \in \{ m, \ldots , m+k \}$. The second derivative of *A* is then prevented from changing abruptly between successive knots. Penalized B-spline estimation is also referred to as P-spline estimation.

## Existence of the B-spline estimator

The procedure described in Sect. 2 is applicable to any order $m \ge 3$ and choice (5) of *k* interior knots, as well as for any smoothing parameter $\lambda _n \in (0, \infty )$. The result below implies that this always leads to at least one minimizer of the map $L : \mathcal {B} \rightarrow [0,\infty )$ in Eq. (8).

### Proposition 1

The set $\mathcal {B}$ is a non-empty, convex, compact subset of $\mathbb {R}^{m+k}$.

### Proof

To see that $\mathcal {B}$ is non-empty, let $\iota = (1, \ldots , 1)$ be a vector of ones and observe that the map $A = \iota ^\top \mathit{\Phi}$ is identically equal to 1 on the interval [0, 1] because the components of $\mathit{\Phi}$ form a partition of unity. It is clear that $A \in \mathcal {A}$ as it corresponds to the independence copula. Therefore, $\iota \in \mathcal {B}$.

That $\mathcal {B}$ is convex and closed is obvious from Conditions (C1)–(C3). To show that $\mathcal {B}$ is also bounded, let $\Vert \cdot \Vert$ be any fixed norm on $\mathbb {R}^{m+k}$. Because the map $\alpha \mapsto \Vert \alpha ^\top \mathit{\Phi} \Vert _\infty$ is continuous, its infimum over the set $\{ \alpha \in \mathbb {R}^{m+k} : \Vert \alpha \Vert =1\}$ is achieved at some $\alpha _*$ therein. As the B-spline basis functions are linearly independent, one has $\Vert \alpha _*^\top \mathit{\Phi} \Vert _\infty = c > 0$. Now for any $\beta \in \mathcal {B}$, one has $\Vert \beta ^\top \mathit{\Phi} \Vert _\infty \le 1$ but also

$$\Vert \beta ^\top \mathit{\Phi} \Vert _\infty =\Vert \beta \Vert \times \bigl \Vert \left( {\beta } / {\Vert \beta \Vert }\right) ^\top \mathit{\Phi} \bigr \Vert _\infty \ge c \, \Vert \beta \Vert ,$$

and hence $\Vert \beta \Vert \le 1/c$. Therefore, $\mathcal {B}$ is bounded. $\Box$

The following result, which will be used in Sect. 4, highlights the fact that the upper bound on the norm of the elements in $\mathcal {B}$ depends on the choice of order $m \ge 3$ but not on the number *k* or location of the interior knots in (5).

### Corollary 1

Given any order $m \ge 2$ and sequence (5) of *k* distinct interior knots, $\sup _{\beta \in \mathcal {B}} \Vert \beta \Vert _\infty \le 2m \,9^{m-1}$.

### Proof

It follows from the proof of Proposition 1 that, for each $\beta \in \mathcal {B}$, one has $\Vert \beta \Vert _\infty \le 1/c$, where

$$c = \inf _{\alpha \in \mathbb {R}^{m+k}, \Vert \alpha \Vert _\infty =1} \Vert \alpha ^\top \mathit{\Phi} \Vert _\infty .$$

Because the *B*-spline basis forms a partition of unity, one has

$$\sup _{\alpha \in \mathbb {R}^{m+k}, \Vert \alpha \Vert _\infty =1} \Vert \alpha ^\top \mathit{\Phi} \Vert _\infty = 1.$$

Indeed, for any vector $\alpha \in \mathbb {R}^{m+k}$ with $\Vert \alpha \Vert _\infty =1$ and any real $t \in [0,1]$,

$$\left| \sum _{j=1}^{m+k}\alpha _j \phi _{j,m}(t)\right| \le \Vert \alpha \Vert _\infty \left\{ \sum _{j=1}^{m+k} \phi _{j,m} (t) \right\} = 1$$

because the basis functions are non-negative and form a partition of unity. This upper bound is attained at $\iota \in \mathbb {R}^{m+k}$. As a consequence, $\sup _{\beta \in \mathcal {B}} \Vert \beta \Vert _\infty \le \kappa _{m,\infty }$, where $\kappa _{m,\infty }$ is the so-called condition number given by

$$\sup _{\ell \ge 0} \sup _{\varvec{\tau }} \frac{\sup _{\alpha \in \mathbb {R}^{m+k}, \Vert \alpha \Vert _\infty =1} \Vert \alpha ^\top \mathit{\Phi} \Vert _\infty }{\inf _{\alpha \in \mathbb {R}^{m+k}, \Vert \alpha \Vert _\infty =1} \Vert \alpha ^\top \mathit{\Phi} \Vert _\infty },$$

where the second supremum is taken over all sequences $\varvec{\tau }$ of knots with $\ell$ distinct interior knots. It was shown by de Boor (1972) that the condition number is at most $2m \,9^{m-1}$, whence the result. $\Box$

## Consistency of the B-spline estimator

Conditions will now be described under which the B-spline estimator defined in Eq. (7) is consistent. To distinguish the true underlying Pickands dependence function from an arbitrary element in the set $\mathcal {A}$, the former will henceforth be denoted $A_0$ and the corresponding extreme-value copula by $C_0$. Other relevant notation is set in Sect. 4.1 and the main result, Theorem 1, is stated in Sect. 4.2. The proof of Theorem 1 per se is relegated to Sect. 5.

### Notation

Fix an order $m \ge 3$ and for each sample size *n*, let $\varvec{\tau }_n = (\tau _1,\ldots , \tau _{2m+k_n})$ be an ordered sequence of knots such that

10 $$\begin{aligned} 0 = \tau _1 = \cdots = \tau _{m}< \tau _{m+1}< \cdots <\tau _{m+k_n+1} = \cdots = \tau _{2m+k_n} = 1. \end{aligned}$$

The notation for the $k_n$ interior knots is consistent with Eq. (5) and the corresponding B-spline basis defined in Eq. (6) can be denoted

$$\mathit{\Phi} _n= (\phi _{n,1,m}, \dots , \phi _{n,m+k_n,m})^\top$$

to emphasize its dependence on *n*.

Similarly, let $\mathcal {B}_n$ denote the set of vectors in $\mathbb {R}^{m+k_n}$ corresponding to the knots (10) and order *m*, as per Eq. (9). Recall that $\mathcal {B}_n$ is a non-empty convex and compact set by Proposition 1, and define $K_n \subset \mathcal {A}$ by

$$K_n = \{ \beta ^\top \mathit{\Phi} _n : \beta \in \mathcal {B}_n\}.$$

For each integer $n \in \mathbb {N}$, now consider the map $L_{0,n}: \mathcal {A} \rightarrow \mathbb {R}$ defined, for each $A \in \mathcal {A}$ such that $\Vert A^{\prime \prime }\Vert _\infty < \infty$, by

11 $$\begin{aligned} L_{0,n}(A) = \frac{1}{n} \big \{\Vert {\hat{Z}} - A ({\hat{T}}) \Vert _1 -\Vert {\hat{Z}}\Vert _1 \big \} + \frac{\lambda _n}{n} \, \Vert A^{\prime \prime }\Vert _\infty , \end{aligned}$$

where $A ({\hat{T}}) = (A({\hat{T}}_1), \ldots , A({\hat{T}}_n))^\top$ and the components of the vectors ${\hat{T}} = ({\hat{T}}_1, \ldots , {\hat{T}}_n)^\top$ and ${\hat{Z}} = ({\hat{Z}}_1, \ldots , {\hat{Z}}_n)^\top$ are as defined in Eq. (4). This map is well-defined on $K_n$ because as stated in Remark 1 in Sect. 5.2,

$$A \in K_n \quad \Rightarrow \quad \Vert A^{\prime \prime }\Vert _\infty < \infty .$$

Clearly, the minimization of $L_{0,n}$ over $K_n$ results in the same estimator as the minimization over $\mathcal {B}_n$ of the objective function *L* in Eq. (8). Therefore, the consistency of the B-spline estimator ${\hat{A}}_n$ can be established by looking at the large-sample behavior of any and all elements in the set

12 $$\begin{aligned} M_n = \left\{ A_n \in K_n: L_{0,n} (A_n) = \min _{A \in K_n} L_{0,n} (A) \right\} . \end{aligned}$$

Note at the outset that $M_n \ne \emptyset$ for every integer $n \in \mathbb {N}$. To this end, endow the set $\mathcal {A}$ with the topology induced by the norm $\Vert \cdot \Vert _\infty$. The objective function $L_{0,n}$ is then continuous and convex on $K_n$, and the latter is a compact set given that it is the image of $\mathcal {B}_n$ with respect to the continuous map $\beta \mapsto \beta ^\top \mathit{\Phi} _n$. Therefore, there exists at least one $\hat{\beta }_n \in \mathcal {B}_n$ such that

$$L_{0,n}(\hat{\beta }_n^\top \mathit{\Phi} _n) = \min _{\beta \in \mathcal {B}_n} L_{0,n}(\beta ^\top \mathit{\Phi} _n).$$

### Statement of the theorem

Assume the following conditions on the sequence $\varvec{\tau }_n$ of knots and on the large-sample behavior of the sequence $\lambda _n$ of smoothing constants.

#### Condition (K)

The sequence $\varvec{\tau }_n$ of knots is such that for some integer $N \in \mathbb {N}$ and all integers $n \ge N$, there exists $\beta _{0,n}\in \mathcal {B}_n$ with

$$\lim _{n \rightarrow \infty } \Vert \beta _{0,n}^\top \mathit{\Phi} _n - A_0 \Vert _\infty = 0.$$

#### Condition (S)

For a given sequence $\varvec{\tau }_n$ of knots with $k_n$ distinct interior knots, $\lambda _n = o(ns_n^2)$, where

$$s_n = \min \big \{ |\tau _{j+1} - \tau _j|: j \in \{ m, \ldots , m+k_n \} \big \} .$$

This paper’s first major finding is then the following.

#### Theorem 1

Fix an order $m \ge 3$ and assume that $\varvec{\tau }_n$ is a sequence of knots fulfilling Condition (K). Suppose also that $\lambda _n$ is a sequence of smoothing constants satisfying Condition (S). For each integer $n \in \mathbb {N}$, let $M_n$ be the set defined in Eq. (12). Then, as $n \rightarrow \infty$,

$$\sup _{A_n \in M_n} \Vert A_n - A_0 \Vert _\infty \overset{p}{\rightarrow } 0,$$

where $A_0$ is the true underlying Pickands dependence function.

When $m \in \{ 3, 4 \}$, it is shown in the Appendix that Condition (K) on the knots is automatically verified for any sequence $\varvec{\tau }_n$ of knots whose mesh size

13 $$\begin{aligned} \epsilon _n = \max \big \{ \tau _{m + i + 1} - \tau _{m + i} : i \in \{ 0, \ldots , k_n \} \big \} \end{aligned}$$

tends to 0 as $n \rightarrow \infty$. The construction described therein could possibly be extended to splines of any order $m \ge 5$. However, the issue is not pursued here, not only because the argument seems involved, but also because the convexity constraints are more difficult to enforce when $m \ge 5$ and this limits the practical use of such spline estimators at present.

Condition (S) regulates the size of the penalty term as $n \rightarrow \infty$. For example, $s_n = 1/(k_n + 1)$ for equidistant interior knots so that if the number $k_n$ of interior knots is of the order of $\sqrt{n}$, say, this condition is then tantamount to requiring that $\lambda _n = o(1)$. It will be shown in Lemma 4 that the penalty term vanishes asymptotically when Condition (S) holds. In their paper, Cormier et al. (2014) adopted the common practice of placing the interior knots on a grid of equally spaced empirical quantiles. Their investigation revealed that taking $k_n \approx \sqrt{n}$ yielded the best performance for the sample sizes in their study. They also considered the use of Schwarz’s information criterion and cross validation techniques to select the penalty term and found that the former was preferable both in terms of performance and computational effort.

These observations motivate the use of the penalty term but as a careful review of the arguments described in Sect. 5 reveals, the conclusions of Theorem 1 remain valid even when $\lambda _n = 0$ for every sample size *n*. That is, the unpenalized version of the estimator is consistent under Condition (K), given that Condition (S) then holds trivially.

## Proof of the consistency result

The argument leading to Theorem 1, which is rather involved, relies on preliminary results reported in Sect. 5.1. Theorem 1 per se is proved in Sect. 5.2.

### Technical preliminaries

Let *C*[0, 1] be the set of continuous functions $f: [0,1] \rightarrow \mathbb {R}$, and endow this space with the uniform norm topology. Let $D_{0,n}$ be the first summand in the definition of the objective function $L_{0,n}$ in Eq. (11). More formally, define the map $D_{0,n}: \mathcal {A} \rightarrow \mathbb {R}$ by setting, for every $A \in \mathcal {A}$,

$$D_{0,n} (A) = \frac{1}{n} \big \{\Vert {\hat{Z}} - A ({\hat{T}}) \Vert _1 -\Vert {\hat{Z}}\Vert _1 \big \} = \frac{1}{n} \sum _{i=1}^n \big \{ |\hat{Z}_i - A (\hat{T}_i)| - |\hat{Z}_i| \big \},$$

where $A ({\hat{T}}) = (A({\hat{T}}_1), \ldots , A({\hat{T}}_n))^\top$ and the components of the vectors ${\hat{T}} = ({\hat{T}}_1, \ldots , {\hat{T}}_n)^\top$ and ${\hat{Z}} = ({\hat{Z}}_1, \ldots , {\hat{Z}}_n)^\top$ are as defined in Eq. (4).

Clearly, the map $D_{0,n}$ is convex. However, it is also Lipschitz with constant 1 given that, for any constant $a \in \mathbb {R}$, the map $x \mapsto |a-x| - |a|$ is itself Lipschitz with the same constant.

It will now be shown that viewed as a function of the random vectors ${\hat{T}}$ and ${\hat{Z}}$, as $n \rightarrow \infty$, $D_{0,n}$ converges in probability, denoted $\overset{p}{\rightarrow }$, to the map $D_{0,\infty }: \mathcal {A} \rightarrow \mathbb {R}$ defined, for all $A \in \mathcal {A}$, by

$$D_{0,\infty } (A) = \mathrm{E} \{| A_0 (T) - A (T) | - | A_0 (T) | \},$$

where *T* denotes the random variable defined as

14 $$\begin{aligned} T = \ln (V) / \ln (UV) \end{aligned}$$

in terms of a random pair (*U*, *V*) distributed as the extreme-value copula $C_0$ with Pickands dependence function $A_0$.

#### Lemma 1

One has, as $n \rightarrow \infty$, $Q_n = \sup _{A \in \mathcal {A}} | D_{0,n}(A) - D_{0,\infty }(A)| \overset{p}{\rightarrow } 0.$

#### Proof

Fix an arbitrary $\delta \in (0, \infty )$. It follows from the Arzelà–Ascoli theorem that the convex set $\mathcal {A}$ is a relatively compact subset of *C*[0, 1]. Given that the set $\mathcal {A}$ is closed, it is actually a compact subset of *C*[0, 1]. One can thus choose a finite set $\{ \alpha _1,\ldots , \alpha _N\}$ of elements of $\mathcal {A}$ such that, for all $A \in \mathcal {A}$, there exists $j(A) \in \{ 1, \ldots , N\}$ such that $\Vert A - \alpha _{j(A)}\Vert _\infty \le \delta$.

From the triangle inequality one has, for any $A \in \mathcal {A}$,

$$\begin{aligned} \begin{aligned} \Vert D_{0,n}(A)& - D_{0,\infty }(A)\Vert \le \Vert D_{0,n}(A) - D_{0,n}(\alpha _{j(A)})\Vert \\ &+ \Vert D_{0,n}(\alpha _{j(A)}) - D_{0,\infty }(\alpha _{j(A)})\Vert + \Vert D_{0,\infty }(\alpha _{j(A)}) - D_{0,\infty }(A)\Vert . \end{aligned} \end{aligned}$$

The first and third terms on the right-hand side are each bounded above by $\delta$ because $D_{0,n}$ and $D_{0,\infty }$ are both Lipschitz with constant 1. Furthermore, the middle term is bounded above by

$$W_n = \max _{j \in \{ 1, \ldots , N \}} | D_{0,n}(\alpha _j) - D_{0,\infty }(\alpha _j) | .$$

Given that $\delta \in (0, \infty )$ is arbitrary, the proof of Lemma 1 will be complete if one can show that, as $n \rightarrow \infty$,

$$W_n \overset{p}{\rightarrow } 0 ,$$

which holds true if, for any fixed $A \in \mathcal {A}$, one has, as $n \rightarrow \infty$,

15 $$\begin{aligned} D_{0,n}(A) - D_{0,\infty }(A) \overset{p}{\rightarrow } 0. \end{aligned}$$

To establish claim (15), define, for any integer $\ell > 2$ and all integers $n \ge \ell$,

$$D_{0,n,\ell } (A) = \frac{1}{n} \sum _{i=1}^n \big \{| {\hat{Z}} _i - A({\hat{T}}_i) | - | {\hat{Z}}_i | \big \} \mathbf {1} \big \{ (\hat{U}_i, \hat{V_i}) \in I_\ell ^2 \big \},$$

where $I_\ell ^2$ stands for the rectangle $[1/\ell , 1-1/\ell ] \times [1/\ell , 1-1/\ell ] \subset [0,1]^2$. Now observe that, for every integer $i \in \{ 1, \ldots , n\}$ and $A \in \mathcal {A}$, one has

16 $$\begin{aligned} \bigl | | {\hat{Z}} _i - A({\hat{T}}_i) | - | {\hat{Z}}_i | \bigr | \le \Vert A \Vert _\infty \le 1. \end{aligned}$$

Further note that using the empirical copula ${\hat{C}}_n$ in Eq. (3), one has

17 $$\begin{aligned} \int _{{\bar{I}}_\ell ^2} \mathrm{d} {\hat{C}}_n (u, v) = \frac{1}{n} \sum _{i=1}^n \mathbf {1} \{ ({\hat{U}}_i, {\hat{V}}_i) \in {\bar{I}}_\ell ^2 \} \le {4}/{\ell } \, , \end{aligned}$$

where ${\bar{I}}_\ell ^2 = [0,1]^2 \setminus I_\ell ^2$ stands for the complement of $I_\ell ^2$ in $[0,1]^2$. Indeed, inequality (17) stems from the fact that

$$\begin{aligned} \#\{ i : ({\hat{U}}_i , {\hat{V}}_i) \in {\bar{I}}_\ell ^2 \}&\le 2\# \{ i: {\hat{U}}_i< 1/\ell \text{ or } {\hat{U}}_i> 1- 1/\ell \}\\&\le 2 \, \# \{ i: i/n < 1/\ell \} + 2 \, \# \{ i: i/n > 1-1/\ell \} \le {4n}/{\ell }. \end{aligned}$$

Combining (16) and (17), one gets

$$|D_{0,n}(A) - D_{0,n,\ell }(A) | \le {4}/{\ell } \, .$$

Next, define

$$D_{0, \infty , \ell }(A) = \mathrm{E} \left[ \left\{ | A(T) - A_0(T)| - |A(T)| \right\} \mathbf {1} \left\{ (u, v)\in I_\ell ^2 \right\} \right]$$

and observe that

$$\begin{aligned} |D_{0,\infty ,\ell }(A)-D_{0,\infty }(A)|&= \mathrm{E}\left[ \left\{ | A_0(T) - A(T)| - |A_0(T)| \right\} \mathbf {1} \left\{ (u, v) \not \in I_\ell ^2 \right\} \right] \\&\le \Vert A\Vert _\infty \Pr \left\{ (u, v) \not \in I_\ell ^2\right\} \\&\le 4 /\ell . \end{aligned}$$

Together with the triangle inequality, the above considerations imply that, for any fixed integer $\ell > 2$ and every integer $n \ge \ell$, one has

18 $$\begin{aligned} \begin{aligned} |D_{0,n}(A) - D_{0,\infty }(A)|&\le |D_{0,n}(A) -D_{0,n,\ell }(A)| + |D_{0,n,\ell }(A) - D_{0,\infty ,\ell }(A)|\\& \quad+|D_{0,\infty ,\ell }(A) - D_{0,\infty }(A)| \\&\le |D_{0,n,\ell }(A) - D_{0,\infty ,\ell }(A)| + 8/\ell . \end{aligned} \end{aligned}$$

It only remains to prove that, as $n\rightarrow \infty$,

19 $$\begin{aligned} |D_{0,n,\ell }(A) - D_{0,\infty ,\ell }(A)| \overset{p}{\rightarrow } 0. \end{aligned}$$

Because the empirical copula ${\hat{C}}_n$ is not strictly positive on its entire domain, it will be convenient to use a slight variant ${\check{C}}_n$ thereof advocated by Bücher et al. (2011), which depends on a constant $\rho \in (1, \infty )$. The exact value of this constant will not have any influence on the proceedings.

For every real $u, v \in (0,1)$, let

20 $$\begin{aligned} {\check{C}}_n (u, v) = \max \{ {\hat{C}}_n (u, v), n^{-\rho } \}. \end{aligned}$$

Because for every integer $i \in \{ 1, \ldots , n\}$, ${\check{C}}_n ({\hat{U}}_i, {\hat{V}}_i) = {\hat{C}}_n ({\hat{U}}_i, {\hat{V}}_i)$, write

$$D_{0,n,\ell }(A) = \int _{I^2_\ell } \left| \frac{\ln \check{C}_n (u, v)}{\ln (uv)} - A \left\{ \frac{\ln (v)}{\ln uv} \right\} \right| - \left| \frac{\ln \check{C}_n (u, v)}{\ln (uv)} \right| d {\hat{C}}_n (u, v)$$

and introduce

$$\begin{aligned} D^*_{0,n,\ell }(A)&= \int _{I^2_\ell } \left| \frac{\ln C_0 (u, v)}{\ln uv} - A \left\{ \frac{\ln (v)}{\ln uv} \right\} \right| - \left| \frac{\ln C_0 (u, v)}{\ln (uv)} \right| d {\hat{C}}_n (u, v) \\&= \int _{I^2_\ell } \left| A_0 \left\{ \frac{\ln (v)}{\ln uv} \right\} - A \left\{ \frac{\ln (v)}{\ln uv} \right\} \right| - \left| A_0 \left\{ \frac{\ln (v)}{\ln uv} \right\} \right| d {\hat{C}}_n (u, v). \end{aligned}$$

Then $$| D_{0,n,\ell }(A) - D^*_{0,n,\ell }(A) | \le O_n(\ell )$$, where

$$O_{n}(\ell )= 2 \sup _{u,v \in I_\ell ^2} \left| \frac{\ln \{\check{C}_n(u, v) /C_0(u, v)\}}{\ln (uv) } \right| = 2 \sup _{u,v \in I_\ell ^2} \left\{ \frac{|\check{C}_n(u, v) -C_0(u, v)|}{|c^*_{u,v}\ln (uv) |} \right\} .$$

The second equality follows from the mean value theorem, which guarantees the existence of the scalar $c^*_{u,v}$ between $C_0(u, v)$ and ${\check{C}}_n(u, v)$.

Given that $c_{u,v}^* \ge \min \{ C_0(u, v),{\check{C}}_n(u, v) \}$, one has

$$O_{n}(\ell ) \le 2 \sup _{u,v \in I_\ell ^2} \left[ \frac{|\check{C}_n(u, v) -C_0(u, v)|}{|\ln (uv) C_0(u, v)|}\max \left\{ 1,\left| \frac{C_0(u, v)}{\check{C}_n(u, v)} \right| \right\} \right] .$$

Now for all $u, v \in I_\ell$, one has

$$- 1/\ln (uv) \le - 1 / \{2\ln (1-1/\ell )\} \quad \text{ and } \quad C_0 (u, v) \ge uv \ge 1/\ell ^2$$

because $C_0$ is an extreme-value copula and hence it is positive quadrant dependent; see, e.g., Genest and Nešlehová (2012). This leads to an upper bound for the right-hand side in the previous display, and hence one gets

$$O_{n}(\ell ) \le \frac{\Vert \check{C}_n - C_0\Vert _\infty }{| 2\ln (1-1/\ell )| (1/\ell ^2)} \times \left[ \sup _{u,v \in I_\ell ^2} \max \left\{ 1,\left| \frac{C_0(u, v)}{\check{C}_n(u, v)} \right| \right\} \right] .$$

Finally, note that $\Vert \check{C}_n - C_0 \Vert _\infty \rightarrow 0$ almost surely, as $n \rightarrow \infty$, by an application of the Glivenko–Cantelli theorem; see, e.g., p. 51 in the monograph by Gänßler and Stute (1987). Consequently, the term in the square brackets converges to 1 almost surely, and, in turn, $O_{n}(\ell ) \rightarrow 0$ almost surely, as $n \rightarrow \infty$. It thus follows that for any integer $\ell > 2$, one has almost surely, as $n \rightarrow \infty$,

$$| D_{0,n,\ell }(A) - D^*_{0,n,\ell }(A) | \rightarrow 0.$$

Furthermore, $D^*_{0,n,\ell }(A)$ is a linear rank statistic with a bounded score function. As such, it converges in probability to $D_{0,\infty ,\ell }(A)$, as $n \rightarrow \infty$; see, e.g., Genest et al. (2013). Thus claim (19) is established; claim (15) then follows from (18). This concludes the proof of Lemma 1. $\square$

The following property of the random variable *T* will prove useful.

#### Lemma 2

If a random pair (*U*, *V*) is distributed according to copula (1) with Pickands dependence function *A*, then the support of the random variable *T* defined in (14) is of the form [*a*, *b*] for some $0 \le a \le 1/2 \le b \le 1$. Moreover, one has $A(t) = 1 - t$ for all $t \in [0, a]$ and $A(t) = t$ for all $t \in [b, 1]$.

#### Proof

Let *F* be the distribution function of *T*. It is known from Proposition 1 in Ghoudi et al. (1998) that for all $t \in (0,1)$, $F(t) = t + t(1-t) A^\prime (t)/A(t)$, where $A^\prime (t)$ denotes the right-hand derivative of *A* at *t*. Now suppose that $F(t) = c \in (0, 1)$ on some interval $I \subset [0, 1]$. As mentioned, e.g., by Capéraà et al. (1997), one must then have, for all $u, v \in I$ with $u \le v$,

21 $$\begin{aligned} \frac{A(v)}{A(u)} = \exp \left\{ \int _u^v \frac{c-t}{t(1-t)} \, \mathrm{d} t \right\} = \frac{v^c (1-v)^{1-c}}{u^c (1-u)^{1-c}}, \end{aligned}$$

which is easily seen to be a non-convex function of *v* for any fixed *u*. This is a contradiction, given that *A* is convex. Therefore, *F* is strictly increasing on the interval $[a, b] \subset [0,1 ]$, where

$$a = \inf \{ t \in [0,1]: F(t) > 0 \}, \quad b = \sup \{ t \in [0, 1]: F(t) < 1 \}.$$

If $a > 0$, then $F(v) = 0$ for all $v \in [0, a)$ and hence it follows from setting $u = 0$ and $c = 0$ in the first equality in Eq. (21) that $A(v) = 1 - v$. Furthermore, one must have $a \le 1/2$, because $1 - a = A(a) \ge 1/2$.

Similarly, if $b < 1$, then $F(u) = 1$ for all $u \in [b, 1]$ and upon setting $v = 1$ and $c = 1$ in the first equality in Eq. (21), one finds that $A(u) = u$. Finally, $b = A(b) \ge 1/2$, thereby concluding the proof of Lemma 2. $\Box$

The following observation concerning $D_{0,\infty }$ will also be needed in the proof of Theorem 1 given in Sect. 5.2.

#### Lemma 3

The unique minimizer of the map $D_{0,\infty }$ over $\mathcal {A}$ is $A_0$.

#### Proof

The map $D_{0,\infty }$ is obviously minimized, over $\mathcal {A}$, at the point $A = A_0$. To show that the minimizer is unique, first assume that $t \in [0,1]$ is in the support of the random variable $T = \ln (V) / \ln (UV)$ with (*u*, *v*) distributed as the extreme-value copula $C_0$ with Pickands dependence function $A_0$. If $A (t) \ne A_0 (t)$, one then has $|A(t) - A_0(t)| > 0$ on some open neighborhood of *t*, which implies that $D_{0,\infty }(A) > D_{0,\infty }(A_0)$. Now it was shown in Lemma 2 that the support of *T* is an interval [*a*, *b*] with $0 \le a \le 1/2 \le b \le 1$ and that for any $t \in [0,1] \setminus [a,b]$, one has $A(t) = \max (t,1-t)$. Therefore, any $A \in \mathcal {A}$ which agrees with $A_0$ on [*a*, *b*] must also agree with $A_0$ on all of [0, 1]. This concludes the proof of Lemma 3. $\square$

### Proof of Theorem 1

For each integer $n \in \mathbb {N}$, let $M_n$ be the set defined in Eq. (12). The fact that this set is nonempty has already been argued at the end of Sect. 4.1. Its convexity follows readily from the convexity of the map $L_{0,n}$. What must be shown is that, as $n \rightarrow \infty$,

$$\sup _{A_n \in M_n} \Vert A_n - A_0 \Vert _\infty \overset{p}{\rightarrow } 0,$$

where $A_0$ is the true underlying Pickands dependence function.

Recall from Lemma 3 that $A_0$ is the unique minimizer of $D_{0,\infty }$ over $\mathcal {A}$. Thus if $A_n$ is any element in the set $M_n$ of minimizers of $L_{0,n}$, one has

22 $$\begin{aligned} L_{0,n}(A_n) \le L_{0,n}(A_{0,n}) \le D_{0,\infty } (A_{0,n}) + Q_n + \frac{\lambda _n}{n} \, \Vert A_{0,n}^{\prime \prime }\Vert _\infty , \end{aligned}$$

where $Q_n$ is as in Lemma 1 and $A_{0,n} = \beta _{0,n}^\top \mathit{\Phi} _n$ is as per Condition (K). The first inequality exploits the fact that $A_{0,n} \in K_n$, while the second holds because $A_{0,n} \in \mathcal {A}$.

Given that $A_0$ is the minimizer of $D_{0,\infty }$ and the latter is Lipschitz with constant 1, one also has

$$D_{0,\infty }(A_{0,n}) - D_{0,\infty }(A_0) = |D_{0,\infty }(A_{0,n}) - D_{0,\infty }(A_0)| \le \epsilon _n,$$

where $\epsilon _n = \Vert A_{0,n}-A_0\Vert _\infty$. By combining this inequality with the chain of inequalities (22), one deduces that

$$D_{0,n}(A_{n}) + \frac{\lambda _n}{n} \, \Vert A_{n}^{\prime \prime }\Vert _\infty - \frac{\lambda _n}{n} \, \Vert A_{0,n}^{\prime \prime }\Vert _\infty - \epsilon _n - Q_n \le D_{0,\infty }(A_0).$$

Again, as $A_0$ is the minimizer of $D_{0,\infty }$, one can conclude that

$$\begin{aligned} 0 \le D_{0,\infty }(A_n) &- D_{0,\infty }(A_0) \le D_{0,\infty }(A_n) - D_{0,n}(A_n) + Q_n + \epsilon _n \\ &- \frac{\lambda _n}{n} \, \Vert A_{n}^{\prime \prime }\Vert _\infty + \frac{\lambda _n}{n} \, \Vert A_{0,n}^{\prime \prime }\Vert _\infty \end{aligned}$$

and hence

23 $$\begin{aligned} 0 \le D_{0,\infty }(A_n) - D_{0,\infty }(A_0)\le 2 Q_n + \epsilon _n + \frac{2\lambda _n}{n} \sup _{\beta \in \mathcal {B}_n} \Vert \beta ^\top \mathit{\Phi} _n^{\prime \prime }\Vert _\infty . \end{aligned}$$

It will now be shown that the third summand in this upper bound, i.e., the penalty term, is asymptotically negligible.

#### Lemma 4

Let $m \ge 3$ be a given order and $\varvec{\tau }_n$ be a sequence of knots with $k_n$ distinct interior knots. If $\lambda _n$ satisfies Condition (S), then, as $n \rightarrow \infty$,

$$\frac{\lambda _n}{n} \, \sup _{\beta \in \mathcal {B}_n} \Vert \beta ^\top \mathit{\Phi} _n^{\prime \prime }\Vert _\infty \rightarrow 0.$$

#### Proof

In view of Condition (S), it suffices to show that $\sup _{\beta \in \mathcal {B}_n} \Vert \beta ^\top \mathit{\Phi} _n^{\prime \prime }\Vert _\infty \le \zeta _m / s_n^2$, where $\zeta _m$ is a constant that depends on *m* but not on *n*. As mentioned on p. 117 of de Boor (2001), one has, for every real $t \in (0,1)$,

$$\beta ^\top \mathit{\Phi} _n^{\prime \prime }(t) = \sum _{j=1}^{m+k} \beta _j^{(3)} \phi _{n,j,m-2}(t),$$

with the convention that when $m=3$, the right-hand second derivative is taken. In the above, $\beta _1^{(3)} = \beta _2^{(3)} = 0$ and, for any integer $j \in \{ 3, \ldots , m+k \}$,

$$\beta _j^{(3)} = \frac{(m-1)(m-2)}{\tau _{j+m-2} - \tau _j} \left( \frac{ \beta _j - \beta _{j-1}}{\tau _{j+m-1} - \tau _j} - \frac{ \beta _{j-1} - \beta _{j-2}}{\tau _{j+m-2} - \tau _{j-1}} \right) .$$

Given that the basis functions $\phi _{n,1,m-2}, \ldots$, $\phi _{n,m+k,m-2}$ form a partition of unity, an argument similar to the proof of Corollary 1 implies that

$$\Vert \beta ^\top \mathit{\Phi} _n^{\prime \prime } \Vert _\infty \le \Vert \beta ^{(3)}\Vert _\infty \le \frac{4 (m-1)(m-2) \Vert \beta \Vert _\infty }{s_n^2} \le \frac{ \zeta _m}{s_n^2}$$

with $\zeta _m = 8m(m-1)(m-2) 9^{m-1}$, where the last inequality follows from Corollary 1 as $\beta \in \mathcal {B}_n$ by assumption. This concludes the proof of Lemma 4. $\Box$

#### Remark 1

The argument developed in the proof of Lemma 4 makes it clear, as already stated in Sect. 4.1, that $A \in K_n \; \Rightarrow \; \Vert A^{\prime \prime }\Vert _\infty < \infty$.

In view of Lemmas 1 and 4, inequality (23) implies

24 $$\begin{aligned} \sup _{A_n \in M_n} | D_{0,\infty }(A_n) - D_{0,\infty }(A_0)| = o_P(1). \end{aligned}$$

Now fix an arbitrary $\eta \in (0, \infty )$. Given that $D_{0,\infty }$ is continuous and that its unique minimizer over $\mathcal {A}$ is $A_0$, one has that, for any fixed integer $n \in \mathbb {N}$,

25 $$\begin{aligned} \inf _{A \in K_n : \Vert A-A_0\Vert _\infty \ge \eta }D_{0,\infty }(A) > D_{0,\infty }(A_0). \end{aligned}$$

Indeed, if this were not the case, one could find a sequence $A_k$ in $K_n$ with $\Vert A_k - A_0\Vert _\infty \ge \eta$ for each $k \in \mathbb {N}$ and $D_{0,\infty } (A_k) \rightarrow D_{0,\infty }(A_0)$, as $k \rightarrow \infty$. Because $K_n$ is compact, however, one could then extract a convergent subsequence converging to $A^* \in K_n$ with the property that $D_{0,\infty }(A^*) = D_{0,\infty }(A_0)$ (by continuity of $D_{0,\infty }$) while at the same time $\Vert A^* - A_0\Vert _\infty \ge \eta$. This would then contradict the fact that $A_0$ is the unique minimizer of $D_{0,\infty }$.

In view of (25), there exists $\xi \in (0, \infty )$ so that $D_{0,\infty } (A) - \xi > D_{0,\infty }(A_0)$ for all $A \in K_n$ with the property that $\Vert A-A_0\Vert _\infty > \eta$. Hence, for any $A \in K_n$,

$$\Vert A - A_0 \Vert _\infty> \eta \quad \Rightarrow \quad |D_{0,\infty }(A) -D_{0,\infty }(A_0)| > \xi .$$

It follows that

$$\Pr \left\{ \sup _{A_n \in M_n} \Vert A_n - A_0\Vert _\infty> \eta \right\} \le \Pr \left\{ \sup _{A_n \in M_n} |D_{0,\infty }(A_n) -D_{0,\infty }(A_0)| > \xi \right\} .$$

The probability on the right-hand side converges to 0, as $n \rightarrow \infty$, by Eq. (24). Because $\eta$ was arbitrary, the proof of Theorem 1 is complete.

## Asymptotic behavior of ${\hat{\beta }}_n$

Having established the existence and consistency of the B-spline estimator (7), the next milestone one would hope to reach is the determination of the asymptotic distribution of this estimator in the broadest possible conditions. At this point, however, this goal remains elusive.

As a step towards a full resolution of this issue, this section describes conditions under which one can identify the limiting distribution of any sequence ${\hat{\beta }}_n$ of minimizers of Eq. (8) involved in the construction of the B-spline estimator of *A*. This result will then be used in Sect. 8 to identify the large-sample distribution of the B-spline estimator ${\hat{A}}_n$.

More specifically, it will be assumed henceforth that the unknown underlying Pickands dependence function $A_0$ is itself a B-spline with a fixed order and a given set of knots. This condition is spelled out below for easy reference.

### Condition (A)

The Pickands dependence function is of the form $A_0 = \beta _0^\top \mathit{\Phi}$ for known knot sequence $\varvec{\tau }$ with *k* internal knots as in (5) and some $m \in \{ 3, 4 \}$. Moreover, the vector $\beta _0$ is in the relative interior of $\mathcal {B}$.

Note that a vector $\beta \in \mathbb {R}^{m+k}$ belongs to the relative interior of $\mathcal {B}$ if and only if (C1) holds and the following conditions, which are stricter than (C2) and (C3), are fulfilled:

(C$$2^\prime$$)
$\beta ^\top \mathit{\Phi} ''(\tau _{m+j}) > 0$ for every integer $j \in \{ 0, \ldots , k + 1\}$.
(C$$3^\prime$$)
$$-1< \beta ^\top \mathit{\Phi} ^\prime (0) < 0$$ and $$0< \beta ^\top \mathit{\Phi} ^\prime (1) < 1$$.

Given the richness of the space spanned by B-splines of orders 3 and 4, including complete freedom in the number and location of internal knots, the assumption that a Pickands dependence function can be written in the form (2) is not a serious limitation in practice, although it implies that the extreme-value copula $C_0$ induced by $A_0$ through Eq. (1) is neither the product copula nor the Fréchet–Hoeffding upper bound, respectively induced by Pickands dependence functions defined, for all $t \in [0,1]$, by $A(t) = 1$ and $A(t) = \max (t, 1-t)$. What is restrictive, however, is the requirement that the set of knots should be known in advance. While this is unrealistic in practice, Theorem 2 below, which is proved in Sect. 7, does represent an essential intermediate step on the way to determining the limiting distribution of $\hat{A}_n$.

Before stating the result, observe that when Condition (A) holds, $C_0$ fulfills Condition 2.1 of Segers (2012). It thus follows from Proposition 3.1 therein that, as $n \rightarrow \infty$, the empirical copula process

$$\mathbb {\hat{C}}_n = \sqrt{n} \, ({\hat{C}}_n - C_0)$$

converges weakly in the space $\ell ^\infty [0,1]^2$ of bounded functions on $[0,1]^2$ equipped with the uniform norm to a $C_0$-pinned centered Gaussian process $\mathbb {\hat{C}}$ defined, for all $u, v \in (0, 1)$, by

$$\mathbb {\hat{C}} (u, v) = \mathbb {C}(u, v) - {\dot{C}}_{0,1} (u, v) \mathbb {C}(u, 1) - {\dot{C}}_{0,2} (u, v) \mathbb {C}(1, v),$$

where, for all real $u, v \in (0,1)$,

$${\dot{C}}_{0,1} (u, v) = \frac{\partial }{\partial u} \, C_0 (u, v) \quad \text{ and } \quad {\dot{C}}_{0,2} (u, v) = \frac{\partial }{\partial v} \, C_0 (u, v),$$

while $\mathbb {C}$ is a Brownian bridge with covariance given, for all $u, v, s, t \in [0,1]$, by

$$\text{ cov } \{ \mathbb {C} (u, v), \mathbb {C} (s,t) \} = C_0 ( u \wedge s, v \wedge t) - C_0 (u, v) C_0 (s, t),$$

with $a \wedge b = \min (a,b)$ for arbitrary reals *a*, $b \in \mathbb {R}$.

### Theorem 2

Let $\lambda _n = o(\sqrt{n})$ and assume Condition (A). Then, as $n \rightarrow \infty$,

26 $$\begin{aligned} {\hat{b}}_n = \sqrt{n} \, ({\hat{\beta }}_n - \beta _0) \rightsquigarrow B = \underset{b \in \mathcal {R}}{\mathrm {argmin}} \left\{ \int _{(0,1)^2} J_b (u, v) \, \mathrm{d} C_0 (u, v) \right\} , \end{aligned}$$

where ${\hat{\beta }}_n$ is any minimizer of (8), $\mathcal {R} =\{0\} \times \mathbb {R}^{m+k-2 }\times \{0\}$, the symbol $\rightsquigarrow$ denotes weak convergence, and, for all $b \in \mathbb {R}^{m+k}$, $u, v \in (0,1)$,

$$J_b (u, v) = \left| \frac{\mathbb {\hat{C}} (u, v)}{\ln (uv) C_0 (u, v)} - b^\top \mathit{\Phi} \left\{ \frac{\ln (v)}{\ln (uv)}\right\} \right| - \left| \frac{\mathbb {\hat{C}} (u, v)}{\ln (uv) C_0 (u, v)}\right| .$$

Furthermore, the argmin in Eq. (26) is almost surely a single point.

Note that the integral in Eq. (26) is finite. Indeed, the elementary inequality $| |x - y|- |x|| \le |y|$ implies that, for all real numbers $u, v \in (0,1)$,

27 $$\begin{aligned} | J_b (u, v)| \le \left| b^\top \mathit{\Phi} \left\{ \frac{\ln (v)}{\ln (uv)} \right\} \right| \le \Vert b \Vert _1 \; \Vert \mathit{\Phi} \Vert _\infty < \infty , \end{aligned}$$

where

$$\Vert \mathit{\Phi} \Vert _\infty = \sup _{j \in \{ 1, \ldots , m+k\}} \sup _{t \in [0,1]} | \phi _{j,m}(t)|.$$

Moreover, it is interesting to note that the limiting distribution defined in Eq. (26) is symmetric about zero. This is formally stated and proved below.

### Proposition 2

The law of *B* is symmetric about zero.

### Proof

Define, for any measurable function *H* on the unit square and vector $b \in \mathbb {R}^{m+k}$, the functional

$$K (H,b) = \int _{(0,1)^2} \left[ \left| H(u, v) - b^\top \mathit{\Phi} \left\{ \frac{\ln (v)}{\ln (uv)} \right\} \right| - |H(u, v)| \right] \mathrm{d} C_0 (u, v).$$

Let $B (H) = \mathrm {argmin}_{b \in \mathcal {R}} \{ K(H,b) \}$. Note that $K(-H,-b) = K(H,b)$ and that $b \in \mathcal {R}$ if and only if $-b \in \mathcal {R}$. Therefore, $B (-H) = - B(H)$.

Now consider the centered Gaussian process defined, for all real numbers $u, v \in (0,1)$, by

$$\mathbb {H} (u, v) = \frac{\mathbb {\hat{C}} (u, v)}{ \ln (uv) C_0 (u, v)} \,.$$

Then because $-\mathbb {H}$ is another Gaussian process with the same law, $B (\mathbb {H})$ and $B (-\mathbb {H})$ are identically distributed, so $- B (\mathbb {H})$ has the same law as $B(\mathbb {H})$. This shows that the law of $B = B (\mathbb {H})$ is symmetric about zero. $\square$

Finally, note that Theorem 2 implies that $\hat{A}_n = \hat{\beta }_n^\top \mathit{\Phi}$ is a consistent estimator of $A_0 = \beta _0^\top \mathit{\Phi}$. However, consistency is actually guaranteed by Theorem 1 under milder assumptions on the tuning parameter $\lambda _n$. Indeed, it is immediate that when Condition (A) holds, Condition (K) is satisfied if $\varvec{\tau }_n=\varvec{\tau }$ for every sample size $n \in \mathbb {N}$. Given that $s_n$ in Condition (S) is then constant, the latter condition is fulfilled whenever $\lambda _n = o(n)$, and this is weaker than $\lambda _n = o(\sqrt{n})$.

As with Theorem 1 the conclusions of Theorem 2 remain valid when the smoothing constant $\lambda _n$ is taken to be equal to zero for every sample size *n*.

## Proof of the limiting distribution of $\hat{\beta }_n$

The proof of Theorem 2 relies on several technical lemmas. An important result about the empirical copula process is first stated in Sect. 7.1; the proof of Theorem 2 is then given in Sect. 7.2.

### Preliminary results concerning the empirical copula process

As in the proof of Lemma 1, the variant ${\check{C}}_n$ of the empirical copula with some tuning constant $\rho \in (1, \infty )$ defined in Eq. (20) will be used. Then, as shown by Bücher et al. (2011), the process

$$\mathbb {\check{C}}_n = \sqrt{n} \, ({\check{C}}_n - C_0)$$

has the same weak limit $\mathbb {\hat{C}}$ as $\mathbb {C}_n$ under Condition 2.1 of Segers (2012), which is met when $A_0$ is continuously differentiable on (0, 1); see Example 5.3 therein. Further recall, as already noted in the proof of Lemma 1, that for every integer $i \in \{ 1, \ldots , n\}$, one has ${\check{C}}_n ({\hat{U}}_i, {\hat{V}}_i) = {\hat{C}}_n ({\hat{U}}_i, {\hat{V}}_i)$ so that the spline estimators of $A_0$ based on $\check{C}_n$ and ${\hat{C}}_n$ coincide.

The following lemma plays an important part in the proof of Theorem 2.

#### Lemma 5

For every integer $\ell > 2$, set $I_\ell = [1/\ell , 1-1/\ell ]$. Then for any extreme-value copula *C* whose Pickands dependence function *A* is continuously differentiable on (0, 1), one has, for $\mathbb {\check{C}}_n = \sqrt{n} \, ({\check{C}}_n - C)$,

$$\sup _{u,v \in I_\ell } \left| \sqrt{n} \; \frac{\ln \{\check{C}_n(u, v)/C(u, v)\} }{\ln (uv)} - \frac{\mathbb {\check{C}}_n(u, v)}{\ln (uv)C(u, v)}\right| = O_P(1/\sqrt{n}).$$

#### Proof

As already noted, if *C* is as specified in the statement of the lemma, the empirical process $\mathbb {\check{C}}_n = \sqrt{n} \, ({\check{C}}_n - C)$ converges weakly, as $n \rightarrow \infty$. Next, from Taylor’s theorem, $\ln (1+x) = x - {x^2}/(2 x_0^2)$ for some $x_0$ between 1 and $1+x$. Thus if for arbitrary reals $u, v \in (0,1)$ one sets $x = {\check{C}}_n(u, v)/C(u, v) - 1$, then

$$\sqrt{n} \; \frac{\ln \{ {\check{C}}_n(u, v) \} - \ln \{C(u, v)\}}{\ln (uv)} = \frac{\mathbb {\check{C}}_n(u, v)}{\ln (uv)C(u, v)} - R_n(u, v),$$

where

$$R_n(u, v) = \frac{1}{2\sqrt{n}}\frac{1}{\ln (uv)} \, \{ \mathbb {\check{C}}_n(u, v) / c^*_{u, v} \}^2$$

and $c^*_{u,v} = x_0 C(u, v)$ is a scalar between *C*(*u*, *v*) and ${\check{C}}_n(u, v)$. Therefore,

$$(c^*_{u,v})^2 \ge \min [ \{ C(u, v)\}^2 , \{ {\check{C}}_n (u, v)\}^2 ],$$

and hence

$$|R_n(u, v)| \le - \frac{1}{2\sqrt{n}}\frac{1}{\ln (uv)} \, \left\{ \frac{\mathbb {\check{C}}_n(u, v)}{C(u, v)} \right\} ^2 \max \left\{ 1, \left| \frac{C(u, v)}{{\check{C}}_n(u, v)} \right| ^2 \right\} .$$

As already argued in the proof of Lemma 1, one has

$$- 1/\ln (uv) \le - 1 / \{2\ln (1-1/\ell )\} \quad \text{ and } \quad C(u, v) \ge uv \ge 1/\ell ^2$$

for all real numbers *u*, $v \in I_\ell$. Moreover, given that the sequence $||\mathbb {\check{C}}_n||_{\infty }$ converges weakly, as $n \rightarrow \infty$, it is uniformly tight and hence

$$\left\{ \frac{\mathbb {\check{C}}_n(u, v)}{C(u, v)} \right\} ^2 \le \left\{ \frac{\Vert \mathbb {\check{C}}_n\Vert _{\infty }}{C(u, v)} \right\} ^2 \le \ell ^4 | |\mathbb {\check{C}}_n||_{\infty }^2 = O_P (1).$$

Finally, note that for all real numbers *u*, $v \in (0,1)$,

28 $$\begin{aligned} \max \left\{ 1, \left| \frac{C(u, v)}{{\check{C}}_n(u, v)} \right| ^2 \right\} \le \sup _{u,v \in I_\ell } \max \left\{ 1, \left| \frac{C(u, v)}{{\check{C}}_n(u, v)} \right| ^2 \right\} . \end{aligned}$$

The right-hand side of (28) converges almost surely to 1, as $n\rightarrow \infty$, for any integer $\ell > 2$, as discussed in the proof of Lemma 1. Thus

$$\sup _{u,v \in I_\ell } | R_n(u, v)| \le -\frac{1}{4 \sqrt{n} \ln (1-1/\ell )} \, O_P(1) = O_P(1/\sqrt{n}),$$

which concludes the proof of Lemma 5. $\Box$

### Proof of Theorem 2

To prove Theorem 2, first introduce, for each integer $i \in \{ 1, \ldots , n\}$, the unobservable random variable

$$\tilde{Z}_i = \frac{\ln \{C_0 (\hat{U}_i,\hat{V}_i)\}}{ \ln (\hat{U}_i \hat{V}_i)} = A_0 ({\hat{T}}_i) = \beta _0^\top \mathit{\Phi} ({\hat{T}}_i).$$

Next set, for all $b \in \mathbb {R}^{m+k}$,

$$D_n (b) = {\left\{ \begin{array}{ll} \displaystyle \frac{1}{n}\sum _{i=1}^n \{ |\sqrt{n} \, ( {\hat{Z}}_i - \tilde{Z}_i) - b^\top \mathit{\Phi} ( {\hat{T}}_i) | - |\sqrt{n} \, ( {\hat{Z}} _i - \tilde{Z}_i) | \} &{} \quad \text {if } b \in \mathcal {B}^*_n, \\ +\infty &{} \quad \text {if } b \not \in \mathcal {B}^*_n, \end{array}\right. }$$

where $\mathcal {B}^*_n$ is the set of vectors $b \in \mathbb {R}^{m+k}$ such that $\beta = \beta _0 + b/\sqrt{n} \in \mathcal {B}$.

Finally, let $P_n$ denote a convex function with values in $[-\infty , +\infty ]$ defined, for all $b \in \mathbb {R}^{m+k}$, by

29 $$\begin{aligned} P_n (b) = D_n (b) + \displaystyle \frac{\lambda _n}{\sqrt{n}} \, \left\| \left( \beta _0 + \frac{b}{\sqrt{n}} \right) ^\top \mathit{\Phi} ^{\prime \prime } \right\| _{\infty } . \end{aligned}$$

With this notation, it is then immediate that

$${\hat{b}}_n = \sqrt{n} \, ({\hat{\beta }}_n - \beta _0) = \text {argmin}_{b \in \mathbb {R}^{m+k}} \{ P_n (b)\}.$$

Therefore, in view of Theorem 3.2 of Geyer (1996), Theorem 2 holds true provided that

(i) $P_n$ epiconverges in law as $n \rightarrow \infty$ to the map *D* given, at any $b \in \mathbb {R}^{m+k}$, by
  $$D(b) = {\left\{ \begin{array}{ll} \displaystyle \int _{(0,1)^2} J_b (u, v) \mathrm{d} C_0(u, v) &{} \text{ if } b \in \mathcal {R}, \\ \infty &{} \text{ if } b \not \in \mathcal {R}, \end{array}\right. }$$
  where $C_0$ is the extreme-value copula induced by $A_0$;
(ii) the minimum of *D* is achieved at a unique point with probability 1.

Each of these conditions will be checked in turn.

#### Epiconvergence in law of $P_n$ to *D*

Given that $\lambda _n = o(\sqrt{n})$ by assumption, the (deterministic) second summand on the right-hand side of Eq. (29) is asymptotically negligible so that, for any fixed vector $b \in \mathbb {R}^{m+k}$,

$$| P_n (b) - D_n (b) | = o(1).$$

By Lemma 3.1 of Geyer (1996) and its preceding discussion, it suffices to show that, for any integer $p \in \mathbb {N}$, the *p*-dimensional distributions of $D_n$ converge in law to those of *D* in the space $[-\infty , \infty ]^p$.

In what follows, the weak convergence of $D_n(b)$ will be shown for given vector $b \in \mathbb {R}^{m+k}$, i.e., as $n \rightarrow \infty$,

30 $$\begin{aligned} D_n (b) \rightsquigarrow D(b). \end{aligned}$$

Higher-dimensional distributions of $D_n$ can then be treated similarly. First note that if $b \not \in \mathcal {R}$, then $D_n (b) = D(b) = + \infty$. If $b \in \mathcal {R}$, then for every sufficiently large integer $n \in \mathbb {N}$,

$$D_n(b) = \int _{(0,1)^2} J_{nb} (u, v) \mathrm{d} {\hat{C}}_n(u, v).$$

where, for all real numbers *u*, $v \in (0,1)$,

$$\begin{aligned} \begin{aligned} J_{nb} (u, v) &= \left| \sqrt{n} \, \frac{\ln \{ \check{C}_n (u, v) / C_0 (u, v)\}}{\ln (uv)} - b^\top \mathit{\Phi} \left\{ \frac{\ln (v)}{\ln (uv)}\right\} \right| \\ &\quad- \left| \sqrt{n} \, \frac{\ln \{ \check{C}_n (u, v) / C_0 (u, v)\}}{\ln (uv)}\right| . \end{aligned} \end{aligned}$$

Next, for every integer $\ell > 2$, set $I_\ell = [1/\ell , 1-1/\ell ]$. Then, for all $n \in \mathbb {N}$,

$$D_{n\ell } (b) = \int _{I_\ell ^2} J_{nb} (u, v) \mathrm{d} {\hat{C}}_n (u, v), \quad D_\ell (b) = \int _{I_\ell ^2} J_b (u, v) \mathrm{d} C_0(u, v).$$

The convergence stated in (30) follows from Wichura’s Theorem (see, e.g., Theorem 4.12 of Billingsley (1968)), provided that, for all vectors $b \in \mathbb {R}^{m+k}$,

31 $$\begin{aligned} \forall _{\ell \in \mathbb {N}, \ell > 2} \quad D_{n\ell }(b) \rightsquigarrow D_\ell (b) \quad \text {as n}\rightarrow \infty , \end{aligned}$$

32 $$\begin{aligned} \forall _{\delta \in (0, \infty )} \quad \lim _{\ell \rightarrow \infty } \limsup _{n \rightarrow \infty } \Pr \{ |D_n(b) - D_{n\ell }(b)| > \delta \} = 0, \end{aligned}$$

33 $$\begin{aligned} \forall _{\delta \in (0, \infty )} \quad \lim _{\ell \rightarrow \infty } \Pr \{ |D_\ell (b) - D(b)| > \delta \} = 0. \end{aligned}$$

To establish (31), introduce, for all reals $u, v \in (0,1)$ and integer $n \in \mathbb {N}$,

$$J_{nb}^* (u, v) = \left| \frac{\mathbb {\check{C}}_n (u, v)}{\ln (uv) C_0 (u, v)} - b^\top \mathit{\Phi} \left\{ \frac{\ln (v)}{\ln (uv)}\right\} \right| - \left| \frac{\mathbb {\check{C}}_n (u, v)}{\ln (uv)C_0(u, v)}\right| .$$

By Lemma 5, $D_{n\ell }(b)$ has the same weak limit as

$$D_{n\ell }^*(b) = \int _{I_\ell ^2} J_{nb}^* (u, v) \mathrm{d} {\hat{C}}_n(u, v).$$

Because, as $n \rightarrow \infty$, $J_{nb}^* \rightsquigarrow J_b$ on the space of bounded functions on $I_\ell ^2 = [1/\ell , 1-1/\ell ] \times [1/\ell , 1-1/\ell ]$ equipped with the uniform norm, it follows from Lemma C8 of Berghaus and Bücher (2017) that, as $n \rightarrow \infty$,

$$D_{n\ell }^*(b) = \int _{I_\ell ^2} J_{nb}^* (u, v) \mathrm{d} {\hat{C}}_n(u, v) \rightsquigarrow D_\ell (b).$$

Turning to (32), one can use the same argument that led to (27) to write

$$|D_{n\ell }(b) - D_n(b)| \le \Vert b \Vert _1 \; \Vert \mathit{\Phi} \Vert _\infty \int _{{\bar{I}}_\ell ^2} \mathrm{d} {\hat{C}}_n(u, v),$$

where ${\bar{I}}_\ell ^2 = (0,1)^2 \setminus I_\ell ^2$. Further recall from Eq. (17) that

$$\int _{{\bar{I}}_\ell ^2} \mathrm{d} {\hat{C}}_n (u, v) \le {4}/{\ell }$$

and that consequently, for any real $\delta \in (0, \infty )$,

$$\Pr \{ |D_n(b) - D_{n\ell }(b)|> \delta \} \le \mathbf {1} \{ 4\Vert b \Vert _1 \; \Vert \mathit{\Phi} \Vert _\infty /\ell > \delta \}.$$

Therefore,

$$\lim _{\ell \rightarrow \infty } \limsup _{n \rightarrow \infty } \Pr \{ |D_n(b) - D_{n\ell }(b)|> \delta \} \le \lim _{\ell \rightarrow \infty } \mathbf {1} \{ 4\Vert b \Vert _1 \; \Vert \mathit{\Phi} \Vert _\infty /\ell > \delta \} =0.$$

Finally, (33) holds because in view of inequality (27), one has

$$|D_\ell (b) - D(b)| \le \int _{{\bar{I}}_\ell ^2} | J_b (u, v) | \mathrm{d} C_0 (u, v) \le \Vert b \Vert _1 \; \Vert \mathit{\Phi} \Vert _\infty \int _{{\bar{I}}_\ell ^2} \mathrm{d} C_0 (u, v),$$

and the right-hand term tends to zero as $\ell \rightarrow \infty$. $\Box$

#### Uniqueness of the minimizer of *D*

Clearly, the set of all minimizers of *D* is convex; it will now be shown that it is non-empty and bounded. Consider the recession function $D_0^+$ of the proper and closed convex function *D* which, by Theorem 8.5 of Rockafellar (1970) and Lebesgue’s dominated convergence theorem, can be written, for $b \in \mathcal {R}$, as

$$D_0^+(b) = \lim _{\lambda \rightarrow \infty } {D(\lambda b)}/{\lambda } = \int _{(0,1)^2} \left| b^\top \mathit{\Phi} \left\{ \frac{\ln (v)}{\ln (uv)} \right\} \right| \mathrm{d} C_0(u, v).$$

For $b \not \in \mathcal {R}$, one has $(D_0^+)(b) = + \infty$.

To prove that $(D_0^+)(b)$ is strictly positive for any non-zero $b \in \mathcal {R}$, note that, for all vectors $b \in \mathcal {R}$,

$$(D_0^+)(b) = \mathrm{E} \{ | b^\top \mathit{\Phi} (T) | \}.$$

So $(D_0^+)(b)$ vanishes if and only if $b^\top \mathit{\Phi} (T) = 0$ almost surely. From Lemma 2, Condition (C$3^\prime$), and the fact that $\mathit{\Phi}$ is continuous, one can then deduce that $b^\top \mathit{\Phi} (t) = 0$ for every real number $t \in [0,1]$. As the components of $\mathit{\Phi}$ are linearly independent, the fact that $b^\top \mathit{\Phi}$ vanishes on [0, 1] implies that $b = 0$.

In view of Corollary 13.3.4(c) in Rockafellar (1970), it thus follows that 0 is an element of the interior of the domain of the convex conjugate $D^*$ of *D*. By Theorem 27.1(d) in that reference, the minimum set of *D*, which is the subgradient $\partial D^*(0)$ of $D^*$ at 0, is non-empty and bounded as asserted.

It will now be argued that $\partial D^*(0)$ is almost surely unique. To this end, consider the process $\mathbb {G}$ defined, for all real numbers *u*, $v \in (0,1)$, by

$$\mathbb {G}(u, v) = \frac{ \mathbb {\hat{C}} (u, v)}{\ln (uv) C_0(u, v)},$$

whose sample paths are continuous on $(0,1)^2$ almost surely. For any such continuous path *G* and vectors *b*, $\nu \in \mathbb {R}^{m+k}$, define

34 $$\begin{aligned} Y = G(u, v) - b^\top \mathit{\Phi} (T), \quad X = \nu ^\top \mathit{\Phi} (T) \end{aligned}$$

and

$$\begin{aligned} K(G, b)&= \int _{(0,1)^2} \left[ \left| G(u, v) - b^\top \mathit{\Phi} \left\{ \frac{\ln (v)}{\ln (uv)} \right\} \right| - |G(u, v) | \right] \, \mathrm{d} C_0(u, v) \\&= \mathrm{E} \big \{ |G(u, v) - b^\top \mathit{\Phi} (T) | - |G(u, v)| \big \} \\&= \mathrm{E} \{ |Y| - |G(u, v)| \}. \end{aligned}$$

It will now be shown that there exists a set $\mathcal{G}$ of continuous paths *G* such that, for each vector $b \in \mathcal {R}$ and vector $\nu \in \mathbb {R}^{m+k}$, $\nu \ne 0$, the map

$$\delta \mapsto L ( \delta ) = \mathrm{E} (| Y - \delta X| - |Y|) = K (G, b + \delta \nu ) + \mathrm{E} \{ |G(u, v)| - |Y|\}$$

is strictly convex on $\mathbb {R}$. From this it then clearly follows that whenever $G \in \mathcal {G}$, the map $b \mapsto K(G, b)$ is strictly convex on $\mathbb {R}^{m+k}$, so that *K*(*G*, *b*) has a unique minimizer in $\mathcal {R}$. It will also be proved hat $\Pr (\mathbb {G} \in \mathcal {G}) = 1$, and hence that $K(\mathbb {G}, b)$ has a unique minimizer in $\mathcal {R}$ almost surely, as claimed. This will rely on the following two technical lemmas.

##### Lemma 6

Suppose that $(W_1, W_2)$ is an arbitrary random pair with support $\mathcal {S}=\mathcal {I} \times \mathbb {R}$, where $\mathcal {I}$ is a closed set such that $\mathcal {I} \ne \{0\}$. Then the map $\delta \mapsto L(\delta ) = \mathrm{E} (| W_2 - \delta W_1| - |W_2|)$ is strictly convex on $\mathbb {R}$.

##### Proof

Without loss of generality, consider $\delta _1 < \delta _2$ and some $\lambda \in (0,1)$. Then

$$\begin{aligned} \lambda L(\delta _1) &+ (1-\lambda )L(\delta _2) - L(\delta ) = \mathrm{E} \left\{ \lambda |W_2 -\delta _1W_1| \right.\\&+\left. (1-\lambda )|W_2-\delta _2 W_1| - |W_2-\delta W_1|\right\} , \end{aligned}$$

where $\delta =\lambda \delta _1+ (1-\lambda )\delta _2$. It turns out that the right-hand side is positive. Indeed, given that the integrand on the right-hand side is non-negative by the triangle inequality, the expectation is positive provided that the integrand is (strictly) positive with non-zero probability. The integrand is positive on the event where the variables $W_2 - \delta _1W_1$ and $W_2 - \delta _2 W_1$ have opposite signs. Because the intersection of the set $\{(w_1,w_2) : (w_2-\delta _1 w_1)(w_2-\delta _2w_1) < 0\}$ and the support $\mathcal {S}$ is relatively open and non-empty, the probability that $(W_1,W_2)$ lies in this intersection is strictly positive. $\Box$

##### Lemma 7

Let $\mathcal{G}$ be the set of all continuous functions on $(0,1)^2$ with the property that for every rational $\gamma \in (0,\infty )$, the map $u \mapsto G(u,u^\gamma )$ is a surjection from (0, 1) to the real line. Then $\Pr (\mathbb {G} \in \mathcal{G}) = 1$.

##### Proof

Fix some rational $\gamma \in (0, \infty )$ and define the map $\varphi :(0,1) \rightarrow (0,1)$ by setting, for every real number $u \in (0,1)$,

$$\begin{aligned} \varphi (u) = C_0(u,u^\gamma )&= \exp \big [ \{(1+\gamma )\ln (u) \} A_0 \{\gamma /(1+\gamma ) \} \big ] \\&= u^{(1+\gamma )A_0 \{\gamma /(1+\gamma ) \}} = u^{\kappa (\gamma )}, \end{aligned}$$

where $\kappa (\gamma ) = (1+\gamma )A_0\{\gamma /(1+\gamma )\}$. Because $A_0$ satisfies Condition (C3$^\prime$), $A_0(t) > \max (t,1-t)$ for every real number $t \in (0,1)$. Consequently,

35 $$\begin{aligned} \kappa (\gamma ) > \max ( \gamma , 1). \end{aligned}$$

It suffices to show that, almost surely,

$$\limsup _{u \downarrow 0} \frac{\hat{\mathbb {C}}(u,u^\gamma )}{(1+\gamma )\varphi (u) \ln (u)} = +\infty , \quad \liminf _{u \downarrow 0} \frac{\hat{\mathbb {C}}(u,u^\gamma )}{(1+\gamma )\varphi (u) \ln (u)} = -\infty .$$

Let $\dot{C}_{0,1}$ and $\dot{C}_{0,2}$ denote the partial derivatives of $C_0$ with respect to its first and second argument, respectively. For every real $u \in (0, e^{-1/\kappa (\gamma )})$, write

$$\frac{\hat{\mathbb {C}}(u,u^\gamma )}{(1+\gamma )\varphi (u) \ln (u)} = \theta (u) \{ N_1(u) - N_2(u) - N_3(u)\},$$

where

$$\theta (u) = \sqrt{\frac{2 \ln \ln \{1/\varphi (u)\}}{(1+\gamma )^2\varphi (u)\ln ^2(u)}}$$

with

$$N_1 (u) = \frac{\mathbb {C}(u,u^\gamma )}{\sqrt{2 \varphi (u) \ln \ln \{1/\varphi (u)\}}}, \quad N_2 (u) = \frac{\dot{C}_{0,1}(u,u^\gamma ) \mathbb {C}(u,1)}{\sqrt{2 \varphi (u) \ln \ln \{1/\varphi (u)\}}},$$

and

$$N_3 (u) = \frac{\dot{C}_{0,2}(u,u^\gamma ) \mathbb {C}(1,u^\gamma )}{\sqrt{2 \varphi (u) \ln \ln \{1/\varphi (u)\}}}.$$

Because $\theta (u) \rightarrow +\infty$ as $u \downarrow 0$, it suffices to investigate the limiting behavior of $N_1$, $N_2$, and $N_3$. Each of these terms is discussed in turn.

(i) *Limiting behavior of* $N_1$. Given that $\varphi$ is strictly increasing, one has
  $$\begin{aligned}\mathrm{cov}\left\{ \mathbb {C} (u,u^\gamma ),\mathbb {C} (v,v^\gamma )\right\} &= \varphi (u\wedge v) -\varphi (u)\varphi (v) \\&= \varphi (u) \wedge \varphi (v) - \varphi (u)\varphi (v),\end{aligned}$$
  so that $\mathbb {C} (u,u^\gamma ) = \mathbb {B}\{\varphi (u)\}$, where $\mathbb {B}$ is a Brownian bridge. Hence
  $$\limsup _{u \downarrow 0} N_1(u) = 1 \quad \text{ and } \quad \liminf _{u \downarrow 0} N_1(u) = -1$$
  almost surely by the law of the iterated logarithm.
(ii) *Limiting behavior of* $N_2$. A straightforward calculation shows that
  $$\begin{aligned}\dot{C}_{0,1}(u,u^\gamma ) = \frac{\varphi (u)}{u} \left\{ A_0\left( \frac{\gamma }{1+\gamma }\right)\right. -\left.\frac{\gamma }{1+\gamma } A_0^\prime \left( \frac{\gamma }{1+\gamma }\right) \right\} \equiv \frac{\varphi (u)}{u} \, c_1(\gamma ).\end{aligned}$$
  Moreover, $\mathbb {C}(u,1)$ is a Brownian bridge. By the law of the iterated logarithm and the fact that $\varphi (u)/u = u^{\kappa (\gamma )-1} \rightarrow 0$ as $u\rightarrow 0$ by (35), one then finds that
  $$\limsup _{u \downarrow 0 } \frac{|\mathbb {C}(u,1)|}{\sqrt{2u \ln \ln (1/u)}}\sqrt{\frac{\varphi (u)}{u}} \sqrt{\frac{ \ln \ln (1/u)}{\ln \ln \{1/\varphi (u)\}}} \, c_1(\gamma ) = 0.$$
(iii) *Limiting behavior of* $N_3$. In this case, note that
  $$\dot{C}_{0,2}(u,u^\gamma ) = \frac{\varphi (u)}{u^\gamma } \left\{ A_0\left( \frac{\gamma }{1+\gamma }\right) +\frac{1}{1+\gamma } A_0^\prime \left( \frac{\gamma }{1+\gamma }\right) \right\} \equiv \frac{\varphi (u)}{u^\gamma } \, c_2(\gamma )$$
  and that $\mathbb {C}(1,u)$ is a Brownian bridge. By the law of the iterated logarithm and the fact that $\varphi (u)/u^\gamma = u^{\kappa ({\gamma })-\gamma } \rightarrow 0$ as $u \rightarrow 0$ by Eq. (35), one finds that
  $$\limsup _{u \downarrow 0 } \frac{|\mathbb {C}(1,u^\gamma )|}{\sqrt{2u^\gamma \ln \ln (1/u^\gamma )}} \sqrt{\frac{\varphi (u)}{u^\gamma }} \sqrt{\frac{ \ln \ln (1/u^\gamma )}{\ln \ln \{1/\varphi (u)\}}} \, c_2(\gamma ) = 0.$$

Put together,

$$\limsup _{u \downarrow 0} \theta (u) \{ N_1(u) - N_2(u) -N_3(u) \} = +\infty$$

and

$$\liminf _{u \downarrow 0} \theta (u) \{ N_1(u) - N_2(u) -N_3(u) \} = -\infty$$

almost surely, as claimed. It follows that this property holds simultaneously across all rational values of $\gamma \in (0, \infty )$, given that this is a countable intersection. This concludes the proof of Lemma 7. $\Box$

To complete the proof of Theorem 2, let $\mathcal {G}$ be as in Lemma 7. By the same result, $\Pr (\mathbb {G} \in \mathcal {G}) =1$. Now pick any element $G \in \mathcal {G}$. The claim follows from Lemma 6 if the support of (*X*, *Y*) given in Eq. (34) is $\mathcal {I} \times \mathbb {R}$, where $\mathcal {I} \ne \{0\}$.

First note that from Lemma 2, the support of *T* is [0, 1] and that one cannot have $\text{ supp } (X) = \mathcal {I} = \{0\}$. For if it were, then $\nu ^\top \mathit{\Phi} (t)$ would be identically 0 in *t* on the interval [0, 1]. But this is only possible if $\nu = 0$ because the elements of $\mathit{\Phi}$ are linearly independent, and $\nu = 0$ is ruled out by assumption.

It remains to check that the support of the pair (*X*, *Y*) is $\mathcal {I} \times \mathbb {R}$. Let $O = (x_1, x_2) \times (y_1, y_2)$ be a relatively open rectangle in $\mathcal {I} \times \mathbb {R}$. The inverse image of $(x_1, x_2)$ under the continuous map $t \mapsto \nu ^\top \mathit{\Phi} (t)$ is open and non-empty so it contains some rational number $t \in (0,1)$. Let $\gamma$ be the rational number $\gamma = t/(1-t)$. Then the range of $G(u, u^\gamma )$ is all of $\mathbb {R}$ so there is a point $u \in (0,1)$ such that $y = G(u, u^\gamma ) - b^\top \mathit{\Phi} (t) \in (y_1, y_2)$. Given that the map

$$(u, v) \mapsto \left( \nu ^\top \mathit{\Phi} \left\{ \frac{\ln (v)}{\ln (uv)} \right\} , G(u, v) - b^\top \mathit{\Phi} \left\{ \frac{\ln (v)}{\ln (uv)} \right\} \right)$$

is continuous, the inverse image of *O* under this map is open in $(0,1)^2$; in particular, it is non-empty.

By Condition (A) and Corollary 4 in Trutschnig et al. (2016), the support of (*U*, *V*) is the unit square and hence every open set has positive measure for the copula $C_0$. Thus

$$\Pr \left\{ Y \in (y_1,y_2), X\in (x_1,x_2)\right\} > 0.$$

Therefore, the support of (*X*, *Y*) satisfies the conditions of Lemma 1. The proof of Theorem 2 is thus complete.

##### Remark 2

Note that the strict inequality constraints imposed in the statement of Theorem 2 on the parameter vector $\beta _0$ can be relaxed. Indeed, the arguments given above extend to the more general case where $\beta _0$ satisfies Conditions (C1)–(C3) provided that $\mathcal {R}$ is redefined as

$$\mathcal {R} = \{b \in \mathbb {R}^{m+k}: \exists _{\epsilon > 0} \; \beta _0 + \epsilon b \in \mathcal{B} \}.$$

This set does not have the property that $b \in \mathcal{B}$ implies that $-b \in \mathcal{B}$, so the symmetry conclusion asserted in Proposition 2 then no longer holds. As an example consider the Pickands dependence function *A* given by

$$A (t) = {\left\{ \begin{array}{ll} 1-t &{} \text{ if } t \in [0,1/4], \\ 3/4 - 2 (t-1/4)(3/4-t) &{} \text{ if } t \in (1/4, 3/4), \\ t &{} \text{ if } t \in [3/4,1]. \end{array}\right. }$$

If both 1/4 and 3/4 are knots, then this map *A* is a B-spline of order 3. In this specific case, the support of *T* is [1/4, 3/4] and the support of the induced extreme-value copula *C* is $\{ (u, v) \in [0,1]^2: u^3 \le v \le u^{1/3}\}$. Small modifications are then required to Lemmas 2 and 7 but the conclusions all hold as given, with $\mathcal {R}$ modified.

## Consequences of Theorem 2

As a first application of Theorem 2, one can now establish the limiting behavior of the empirical process defined, for all $t \in [0,1]$, by

$$\mathbb {A}_n (t) = \sqrt{n} \, \{ {\hat{A}}_n (t) - A_0 (t) \},$$

where ${\hat{A}}_n$ is as defined in Eq. (7). Because $\Vert \mathit{\Phi} \Vert _\infty < \infty$, the map $\mathbb {R}^{m+k} \rightarrow \ell ^\infty [0,1]$ given by $\beta \mapsto \beta ^\top \mathit{\Phi}$ is continuous. Theorem 2, the continuous mapping theorem, and Proposition 2 thus together imply the following result.

### Corollary 2

Suppose that Condition (A) holds and that $\lambda _n = o(\sqrt{n})$. Then, as $n \rightarrow \infty$, $\mathbb {A}_n \rightsquigarrow B^\top \mathit{\Phi}$ in $\ell ^\infty [0,1]$. In particular, the estimator ${\hat{A}}_n$ is consistent and asymptotically unbiased.

Beyond being intrinsic, the estimator ${\hat{A}}_n$ has the advantage of being smooth and its derivatives are easy to compute. The latter are of interest as they can be used to construct estimators of the spectral distribution *S* associated with *A* and its density *s*, whenever it exits. The spectral density is particularly appealing in practice, because it provides greater visual insight than *A* into the nature of the dependence between *X* and *Y*.

Given a Pickands dependence function *A*, the corresponding unique spectral distribution function *S* on the interval [0, 1] is implicitly defined via

36 $$\begin{aligned} A(t) = 2 \int _0^1 \max \{ (1-w)t, w(1-t) \} \mathrm{d} S(w) = 1 - t + 2 \int _0^t S(w) dw, \end{aligned}$$

which is valid for every real number $t \in [0,1]$; see Einmahl and Segers (2009). From Eq. (36), one can easily deduce that, for every real number $t \in [0,1)$,

$$S(t) = \frac{1}{2} \,A^\prime (t) + \frac{1}{2} \, ,$$

where $A^\prime$ denotes the right-hand derivative of *A*, while of course $S(1) = 1$. If the map $A^\prime$ is absolutely continuous on (0, 1), then $S = S_1 + S_2$, where $S_1$ is discrete with support $\{0,1\}$ and $S_2$ is absolutely continuous with density given, for almost all real numbers $t \in (0,1)$, by $s(t) = A^{\prime \prime }(t) /2$.

When $A = \beta _0^\top \mathit{\Phi}$ and $m\in \{3,4\}$, the map $A^\prime = \beta _0^\top \mathit{\Phi} ^\prime$ is absolutely continuous on (0, 1) with derivative $\beta _0^\top \mathit{\Phi} ^{\prime \prime }$ wherever it exists. Hence, for every real number $t \in [0,1)$, one has

37 $$\begin{aligned} S(t) = \frac{1}{2} \, \beta _0^\top \mathit{\Phi} ^\prime (t) + \frac{1}{2} = \frac{1}{2} \, \sum _{i=1}^{m+k} \beta _{0,i} \phi ^\prime _{i,m}(t) + \frac{1}{2} \end{aligned}$$

while for any real $t \in (0,1)$,

38 $$\begin{aligned} s(t) = \frac{1}{2} \, \beta _0^\top \mathit{\Phi} ^{\prime \prime }(t) = \frac{1}{2} \, \sum _{i=1}^{m+k} \beta _{0,i} \phi ^{\prime \prime }_{i,m}(t), \end{aligned}$$

where the $\phi ^{\prime \prime }$ is understood as the right-hand derivative when $m = 3$.

One advantage of working with B-splines is that the derivatives of the basis functions are readily available through a recursive formula valid for every integer $j \in \{ 1, \ldots , k+2m -\ell \}$ and real $t \in [0, 1]$, viz.

$$\frac{d}{dt} \, \phi _{j,\ell } (t) = \frac{\ell -1}{\tau _{j+\ell -1} - \tau _j} \, \phi _{j,\ell -1} (t) - \frac{\ell -1}{\tau _{j+\ell } - \tau _{j+1}} \, \phi _{j+1,\ell -1} (t).$$

Consequently, $A^\prime$ and $A^{\prime \prime }$ are linear combinations of B-splines of order $m-1$ and $m-2$, respectively, on the original knot vector with a new set of weights.

In view of Eqs. (37) and (38), it makes sense to estimate *S* and *s* by replacing $\beta _0$ by ${\hat{\beta }}_n$ in these formulas, i.e., for every real $t \in [0,1)$,

39 $$\begin{aligned} \hat{S}_n(t) = \frac{1}{2} \, {\hat{\beta }}_n^\top \mathit{\Phi} ^\prime (t) + \frac{1}{2}\, , \quad \hat{s}_n(t) = \frac{1}{2} \, {\hat{\beta }}_n^\top \mathit{\Phi} ^{\prime \prime }(t), \end{aligned}$$

while $\hat{S}_n(1) =1$. As before, $\mathit{\Phi} ^\prime (0)$ and $\mathit{\Phi} ^{\prime \prime }(t)$ for any real $t \in [0,1)$ are right-hand derivatives, while $\mathit{\Phi} ^{\prime \prime }(1)$ is a left-hand derivative.

Because $\Vert \mathit{\Phi} ^\prime \Vert _\infty < \infty$ and $\Vert \mathit{\Phi} ^{\prime \prime }\Vert _\infty < \infty$, the following are straightforward consequences of Theorem 2 and the continuous mapping theorem.

### Corollary 3

Suppose that Condition (A) holds and that $\lambda _n = o(\sqrt{n})$. Let $\tilde{S} = S$ and $\tilde{S}_n = \hat{S}_n$ on [0, 1) while $\tilde{S}(1) = S(1-)$ and $\tilde{S}_n(1) = \hat{S}_n(1-)$. Then, as $n \rightarrow \infty$, $\sqrt{n} \, (\tilde{S}_n - \tilde{S}) =$ $\hat{b}_n^\top \mathit{\Phi} ^\prime /2 \rightsquigarrow B^\top \mathit{\Phi} ^\prime /2$ in $\ell ^\infty [0,1]$, with the convention that $\mathit{\Phi} ^\prime$ at 0 and 1 is the respective one-sided derivative.

### Corollary 4

Suppose that Condition (A) holds and that $\lambda _n = o(\sqrt{n})$. Then, as $n \rightarrow \infty$, $\sqrt{n} \, (\hat{s}_n - s) =\hat{b}_n^\top \mathit{\Phi} ^{\prime \prime }/2 \rightsquigarrow B^\top \mathit{\Phi} ^{\prime \prime }/2$ in $\ell ^\infty [0,1]$.

It follows from Corollary 3, Corollary 4, and Proposition 2 that the estimators ${\hat{S}}_n$ and ${\hat{s}}_n$ are consistent and asymptotically unbiased. Note also that because ${\hat{A}}_n$ is intrinsic, $\hat{S}_n$ is intrinsic as well, i.e., it satisfies the moment constraint that the expected value of the random variable with distribution function $\hat{S}_n$ equals 1/2.

**Fig. 2:**
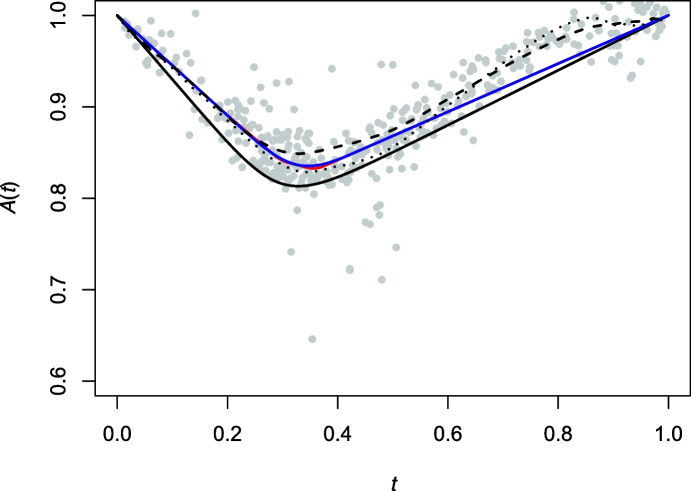
*A*-plot of a sample of size 400 from Gumbel’s asymmetric logistic copula model with $\alpha = .7$, $\beta = .3$ and $\theta = 6$, along with the true Pickands dependence function (in black), the rank-based end-point corrected Pickands (dotted) and CFG (dashed) estimators, and the B-spline estimators with the same set of knots when $m=3$ (in red) and $m=4$ (in blue)

To illustrate, consider a random sample of size $n = 400$ from Gumbel’s asymmetric logistic copula model with $\alpha = .7$, $\beta = .3$, and $\theta = 6$ (Tawn 1988). Figure 2 displays the *A*-plot of the sample along with the true Pickands dependence function in black. Also plotted on this graph are:

- the rank-based, end-point corrected, Pickands (dotted) and so-called CFG (dashed) estimators from Genest and Segers (2009);
- the rank-based B-spline estimators of degree $m = 3$ (in red) and $m = 4$ (in blue) investigated herein.

The B-spline estimators were computed using 40 internal knots; the penalties were chosen to be $\lambda = 15$ when $m = 3$ and $\lambda = 70$ when $m = 4$, based on the cross-validation procedure described by Cormier et al. (2014).

Looking at Fig. 2, one can see that although the Pickands and Capéraà–Fougères–Genest (CFG) estimators meet the end-point constraints, they are not convex, and hence not intrinsic. In contrast, the B-spline estimators are valid Pickands dependence functions by design. The graphs of the curves corresponding to B-splines of degree 3 and 4 are nearly superposed, showing little improvement when the degree is increased from 3 to 4.

**Fig. 3:**
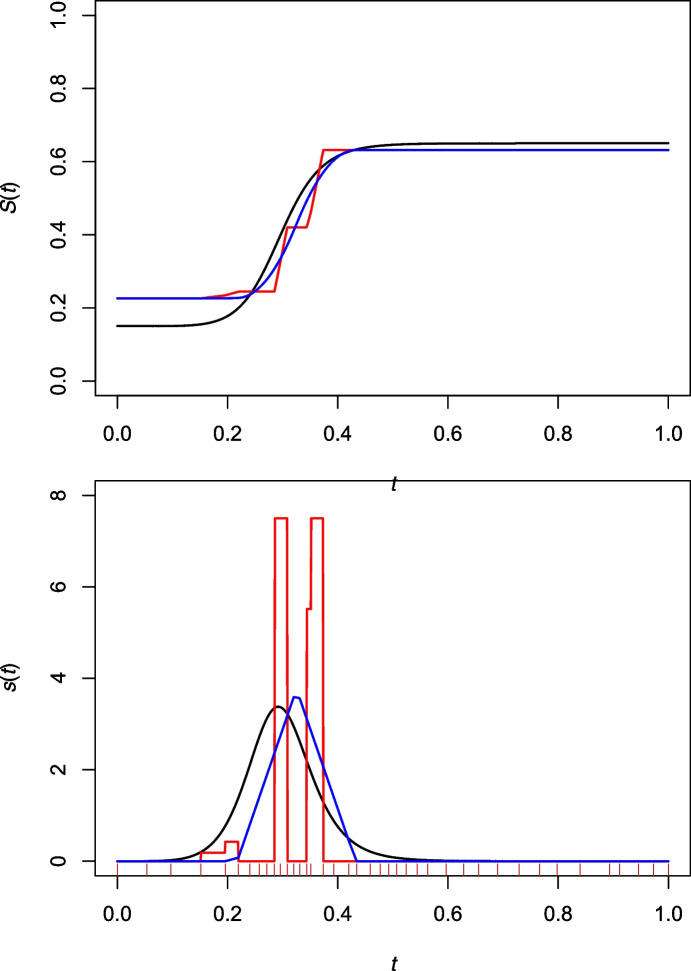
Graphs (in black) of the spectral distribution (top) and density (bottom) corresponding to Gumbel’s asymmetric logistic model with parameters $\alpha = .7$, $\beta = .3$ and $\theta = 6$, along with estimates thereof based on B-splines of order $m = 3$ (in red) and $m=4$ (in blue) with the same set of knots positioned as marked on the horizontal axis in the lower panel

While the degree of the B-splines may not matter much when estimating *A*, it has a perceptible effect when estimating *S*, and even more so when estimating *s*. This is portrayed in Fig. 3, which is based on the same data as Fig. 2. The left panel shows the estimators ${\hat{S}}_n$ of *S* in Eq. (39) corresponding to B-splines estimators of degree 3 (in red) and 4 (in blue). The latter is distinctly superior to the former, although both perform very similarly in estimating the point masses at 0 and 1, whose theoretical values are .15 and .35, respectively.

The right panel of Fig. 3 shows the corresponding estimators of *s*. Clearly, the estimator ${\hat{s}}_n$ in Eq. (39) based on B-splines of degree 4 is far superior to the analogous estimator constructed from B-splines of degree 3. The horizontal axis of this graph also reports the position of the 40 internal knots, which correspond to quantiles of the empirical distribution of the set $T_1, \ldots , T_{400}$, as recommended by Cormier et al. (2014).

## Conclusion

This paper examined the large-sample behavior of a rank-based procedure proposed by Cormier et al. (2014) for the intrinsic estimation of the Pickands dependence function characterizing a bivariate extreme-value copula. The estimator, which involves a linear combination of B-splines of order $m \ge 3$, was shown to be consistent under Conditions (K) and (S), which specify the large-sample behavior of the set of knots and of the penalty term, respectively.

This consistency result, Theorem 1, suggests that it should be possible to determine the asymptotic distribution of this estimator under appropriate conditions. This seems difficult, however. As a partial solution to this problem, the large-sample distribution of the estimator was computed under the assumption that the underlying Pickands dependence function, *A*, can be expressed as a linear combination of *B*-spline basis elements of a given order $m \in \{ 3, 4 \}$ with a fixed and known set of knots. Given the richness of quadratic and cubic B-splines, the condition on *m* is not much of a limitation, but knowledge of the number and position of the interior knots is a practically unrealistic assumption which will hopefully be lifted in subsequent work.

In the future, it would also be worth investigating how one could relax the requirement that $m \in \{ 3, 4 \}$. A visual motivation for this extension is provided in the bottom panel of Fig. 3. The difficulty in this endeavor is the convexity constraint, which is no longer simple to formulate when $m > 4$.

Finally, there would be merit in comparing the performance of the estimators of *S* and *s* proposed herein to other nonparametric estimators of the same quantities considered in the growing literature on the subject. Relevant references include Einmahl et al. (2001), Einmahl and Segers (2009), Guillotte et al. (2011), de Carvalho et al. (2013), as well as the Bayesian estimation method based on normalized B-splines of Khadraoui and Ribereau (2019).

## Data Availability

The dataset used to produce Fig. 2 was generated using the rCopula function from the R copula package. The code is available from the corresponding author upon reasonable request.

## Appendix

The purpose of this Appendix is to show that when $m \in \{ 3, 4 \}$, Condition (K) is automatically satisfied for knot sequences as in (10) whose mesh size $\epsilon _n$ given by (13) tends to 0 as $n \rightarrow \infty$. That is, it will be shown that for these values of *m*, one can always find a sequence $\beta _{0,n}$ of vectors in $\mathcal {B}_n$, such that

40 $$\begin{aligned} \lim _{n \rightarrow \infty } \Vert \beta _{0,n}^\top \mathit{\Phi} _n - A_0 \Vert _\infty = 0. \end{aligned}$$

To this end, a map $A_{0,n} : [0,1] \rightarrow [1/2,1]$ will be constructed so that

(i) $A_{0,n}$ is a polynomial of order at most $m-1$ on the interval $[\tau _{m+i}, \tau _{m+i+1}]$ for each integer $i \in \{ 0, \ldots , k_n \}$;
(ii) $A_{0,n}$ is $(m-2)$-times continuously differentiable on (0, 1);
(iii) $$A_{0,n} \in \mathcal {A}$$;
(iv) $$\Vert A_0 - A_{0,n} \Vert _\infty \le 2^{m-1} \epsilon _n$$.

When $A_{0,n}$ satisfies (i)–(iii), the Curry–Schoenberg Theorem (Curry and Schoenberg 1966), also reported as Theorem (44) by de Boor (2001), then implies the existence of $\beta _{0,n}\in \mathcal {B}_n$ such that $A_{0,n} = \beta _{0,n}^\top \mathit{\Phi} _n$. Property (iv) then yields that (40) holds and therefore the knot sequence $\varvec{\tau }_n$ fulfills Condition (K) as soon as $\epsilon _n \rightarrow 0$ as $n \rightarrow \infty$.

Before examining the cases $m = 3$ and $m = 4$ separately, recall that the right-hand derivative $A_0^\prime$ of $A_0 \in \mathcal {A}$ is monotone increasing given that $A_{0}$ is convex by assumption.

### *Case*$m = 3$

Define two splines, $\varphi ^-$ and $\varphi ^+$, of order 2, i.e., continuous piece-wise linear, as follows. For each integer $i \in \{ 0, \ldots , k_n + 1 \}$, set

$$\varphi ^- (\tau _{m+i}) = A_0^\prime (\tau _{m + i - 1}) \quad \text {and} \quad \varphi ^+ (\tau _{m+i}) = A_0^\prime (\tau _{m + i + 1}).$$

Between knots, define both functions $\varphi ^-$ and $\varphi ^+$ by linear interpolation. Finally, extend the definition of $A_0^\prime$ by putting $A_0^\prime (t) = A_0^\prime (0)$ for every real $t \in (-\infty , 0)$ and $A_0^\prime (t) = A_0^\prime (1)$ for every real $t \in (1, \infty )$.

It is easy to see that, for every real $t \in (0, 1)$, one has

$$A_0^\prime (t - 2\epsilon _n) \le \varphi ^- (t) \le A_0^\prime (t) \le \varphi ^+ (t) \le A_0^\prime (t + 2\epsilon _n).$$

This string of inequalities may be integrated to get

$$\begin{aligned} \int _0^1 A_0^\prime (t) dt -\int _{1 - 2\epsilon _n}^1 A_0^\prime (t) \, dt + 2\epsilon _n A_0^\prime (0) & \le \int _0^1 \varphi ^- (t) dt \le \int _0^1 A_0^\prime (t) dt \le \int _0^1 \varphi ^+ (t) dt \\& \le \int _0^1 A_0^\prime (t) dt + 2\epsilon _n A_0^\prime (1) -\int _0^{2\epsilon _n} A_0^\prime (t) dt. \end{aligned}$$

The monotonicity of $A_0^\prime$ then implies that

41 $$\begin{aligned} \begin{aligned} \int _0^1 A_0^\prime (t) dt - 2 \epsilon _n \{A_0^\prime (1) - A_0^\prime (0)\} & \le \int _0^1 \varphi ^- (t) dt \le \int _0^1 A_0^\prime (t) dt \le \int _0^1 \varphi ^+ (t)dt \\ &\le \int _0^1 A_0^\prime (t) dt + 2 \epsilon _n \{A_0^\prime (1) - A_0^\prime (0) \}. \end{aligned} \end{aligned}$$

In view of the central inequalities in the chain (41), there must exist a real number $\lambda \in [0,1]$ with

$$\int _0^1 \varphi _\lambda (t) dt = \int _0^1 A_0^\prime (t) dt = A_0 (1) - A_0 (0) = 0,$$

where $\varphi _\lambda = \lambda \varphi ^- + (1 - \lambda ) \varphi ^+$. Notice that $\varphi _\lambda$ is a continuous, monotone increasing, piece-wise linear spline with the given set of knots. It follows that the map defined, for every real $t \in [0, 1]$, by

$$A_{0,n} (t) = A_0 (0) + \int _0^t \varphi _\lambda (x) dx = 1+\int _0^t \varphi _\lambda (x) dx$$

fulfills conditions (i) and (ii). The fact that (iii) holds is an immediate consequence of Theorem 1 in Trutschnig et al. (2016). Finally, it follows from the string of inequalities (41) that, for every real $t \in [0,1 ]$, one has

$$| A_0 (t) - A_{0,n} (t) | \le 2 \epsilon _n \{A_0^\prime (1) - A_0^\prime (0)\} \le 4 \epsilon _n,$$

which establishes (iv) and thus the required claim.

### *Case*$m = 4$

First, focus temporarily on a subsequence of knots given by

42 $$\begin{aligned} 0= \tau _m< \tau _{m + 2}< \cdots< \tau _{m + k_n - 1} < \tau _{m + k_n + 1} = 1 \end{aligned}$$

when $k_n$ is odd and by

43 $$\begin{aligned} 0=\tau _m< \tau _{m + 2}< \cdots< \tau _{m + k_n } < \tau _{m + k_n + 2} = 1 \end{aligned}$$

when $k_n$ is even. Now proceed as in the case $m = 3$ to construct continuous piece-wise linear maps $\varphi ^-$ and $\varphi ^+$ on this subsequence of knots. This means that for each integer $i \in \{ 0, \ldots , \lfloor k_n/2 \rfloor +1 \}$,

$$\varphi ^- (\tau _{m+2i}) = A_0^\prime (\tau _{m + 2i - 2}) \quad \text {and} \quad \varphi ^+ (\tau _{m+2i}) = A_0^\prime (\tau _{m + 2i + 2}),$$

As when $m = 3$, extend the definition of $A_0^\prime$ by putting $A_0^\prime (t) = A_0^\prime (0)$ for every real $t \in (-\infty , 0)$ and $A_0^\prime (t) = A_0^\prime (1)$ for every real $t \in (1, \infty )$. One then has, for every real $t \in (0, 1)$,

$$A_0^\prime (t - 4\epsilon _n) \le \varphi ^- (t) \le A_0^\prime (t) \le \varphi ^+ (t) \le A_0^\prime (t + 4\epsilon _n).$$

Next, construct quadratic splines $\psi ^-$ and $\psi ^+$ defined on the original set of knots which are monotone and interpolate $\varphi ^-$ and $\varphi ^+$ at the subset of knots in (42) or (43), as follows.

When $k_n$ is odd, define $\psi ^+$ on the interval $[\tau _{m + 2i}, \tau _{m + 2 (i+1)}]$ for any integer $i \in \{ 0, \ldots , (k_n - 1)/2 \}$, by letting

$$\psi ^+ (t) = {\left\{ \begin{array}{ll} \varphi ^+ (\tau _{m+2i}) + a_{i+1} (t - \tau _{m + 2i})^2 &{} \text{ if } t \in [\tau _{m + 2i}, \tau _{m + 2i + 1}], \\[2mm] \varphi ^+ (\tau _{m + 2i + 2}) - b_{i+1} (t - \tau _{m + 2i + 2})^2 &{} \text{ if } t \in (\tau _{m + 2 i + 1}, \tau _{m + 2i + 2}], \end{array}\right. }$$

where the reals $a_{i+1}$ and $b_{i+1}$ are chosen so that the map $\psi ^+$ is continuously differentiable at $\tau _{m+2i+1}$. Notice that the derivative of $\psi ^+$ is 0 at both $\tau _{m+2i}$ and $\tau _{m+2i+2}$ and that if continuous differentiability at $\tau _{m+2i+1}$ is satisfied, then the result is monotone over $[\tau _{m + 2i}, \tau _{m + 2i +2}]$. This leads to the constraints

$$\begin{aligned} \varphi ^+ (\tau _{m+2i}) &+ a_{i+1} (\tau _{m+2i+1} - \tau _{m+2i})^2 = \varphi ^+ (\tau _{m+2i+2})\\& - b_{i+1} (\tau _{m+2i+1} - \tau _{m+2i+2})^2 \end{aligned}$$

and

$$2a_{i+1} ( \tau _{m+2i+ 1} - \tau _{m+2i} ) = -2 b_{i+1} (\tau _{m+2i+1} - \tau _{m+2i+2}).$$

These constraints are satisfied by

$$a_{i+1} = \frac{\varphi ^+ (\tau _{m+2i+2}) - \varphi ^+ (\tau _{m+2i})}{(\tau _{m+2i+2} - \tau _{m+2i}) (\tau _{m+2i+1} - \tau _{m+2i})}$$

and

$$b_{i+1} = \frac{\varphi ^+ (\tau _{m+2i+2}) - \varphi ^+ (\tau _{m+2i}) }{(\tau _{m+2i+2} - \tau _{m+2i}) (\tau _{m+2i+2} - \tau _{m+2i+1})} \, .$$

When $k_n$ is even, define $\psi ^+(t)$ on $[\tau _{m + 2i}, \tau _{m + 2 (i+1)}]$ for any integer $i \in \{ 0, \ldots , k_n/2 -1\}$ the same way as when $k_n$ is odd. Then for any real $t \in [\tau _{m+k_n}, \tau _{m+k_n+2}]$, set

$$\psi ^+(t) = \varphi ^+(\tau _{m+k_n}) + \frac{\varphi ^{+}(\tau _{m+k_n+2}) - \varphi ^{+}(\tau _{m+k_n})}{(\tau _{m+k_n+2} - \tau _{m+k_n})^2} \, (t-\tau _{m+k_n})^2.$$

The resulting map $\psi ^+$ is again continuously differentiable on (0, 1).

In view of this construction and an entirely analogous one for $\psi ^-$, one has, for every real $t \in (0, 1)$,

$$A_0^\prime (t - 4\epsilon _n) \le \psi ^- (t) \le A_0^\prime (t) \le \psi ^+ (t) \le A_0^\prime (t + 4\epsilon _n).$$

Now, proceeding along similar lines as in the case $m = 3$, one finds that

44 $$\begin{aligned} \begin{aligned} \int _0^1 A_0^\prime (t) dt - 4 \epsilon _n \{A_0^\prime (1) - A_0^\prime (0)\} & \le \int _0^1 \psi ^- (t) dt \le \int _0^1 A_0^\prime (t) dt \le \int _0^1 \psi ^+ (t)dt\\ &\le \int _0^1 A_0^\prime (t) dt + 4 \epsilon _n \{A_0^\prime (1) - A_0^\prime (0) \}. \end{aligned} \end{aligned}$$

Therefore, one can find a scalar $\lambda \in [0, 1]$ such that

$$\int _0^1 \psi _\lambda (t) dt = \int _0^1 A_0^\prime (t) dt = A_0 (1) - A_0 (0) = 0,$$

where the map $\psi _\lambda = \lambda \psi ^- + (1 - \lambda ) \psi ^+$ is a continuously differentiable, monotone increasing, piece-wise quadratic spline with the given set of knots. Arguing analogously as in the case $m=3$ and using Theorem 1 in Trutschnig et al. (2016), one finds that the map defined, for every real $t \in [0, 1]$, by

$$A_{0,n} (t) = A_0 (0) + \int _0^t \psi _\lambda (x) dx = 1+\int _0^t \psi _\lambda (x) dx$$

satisfies (i)–(iii). Finally, the inequalities (44) yield

$$\Vert A_0 - \phi \Vert _\infty \le 4 \epsilon _n \{A_0^\prime (1) - A_0^\prime (0)\} \le 8 \epsilon _n,$$

so that (iv) holds as well, and this concludes the argument.
